# 8,9-Dihydrocannabidiol, an Alternative of Cannabidiol, Its Preparation, Antibacterial and Antioxidant Ability

**DOI:** 10.3390/molecules28010445

**Published:** 2023-01-03

**Authors:** Qi Wu, Maoyue Guo, Lianghua Zou, Qiqi Wang, Yongmei Xia

**Affiliations:** 1 State Key Laboratory of Food Science and Technology, Jiangnan University, Wuxi 214122, China; 2 School of Chemical and Material Engineering, Jiangnan University, Wuxi 214122, China; 3 School of Life Science and Health Engineering, Jiangnan University, Wuxi 214122, China

**Keywords:** 8,9-dihydrocannabidiol, cannabidiol, antimicrobial, antioxidation, fibroblasts

## Abstract

Cannabidiol (CBD) from *Cannabis sativa* is used in cosmetics in North America due to its antibacterial and antioxidant properties, but has been prohibited in many countries except recently; so, finding a non-intoxicating CBD alternative and elucidating the structure–function relationship of CBD analogues is becoming increasingly relevant. Herein, a set of CBD analogues including 8,9-dihydrocannabidiol (H_2_CBD) was synthesized, and their antibacterial, bactericidal, and antioxidant activity, as well as their structure–function relationship, were studied. The results present a catalytic selectivity near 100% towards H_2_CBD with a production yield of 85%. Each CBD analogue presented different antibacterial and antioxidant activity. It is revealed that the phenolic hydroxyl moiety is an essential group for CBD analogues to perform antibacterial and antioxidant activities. Among them, H_2_CBD presented much stronger antibacterial activity than the assayed popular antibiotics. H_2_CBD and Compound 4 presented very similar radical scavenging activity and inhibition on lipid oxidation to vitamin C, but better thermostability. Moreover, H_2_CBD presented lower toxicity to human skin fibroblasts at concentrations up to 64-fold higher than its MIC value (1.25 μg/mL) against *S. aureus.* Above all, in all property experiments, H_2_CBD presented extremely similar performance to CBD (*p* < 0.05), including similar time–kill kinetics curves. This research finds H_2_CBD to be an alternative for CBD with very high potential in the aspects of antibacterial, bactericidal, and antioxidant activity, as well as lower toxicity to human skin fibroblasts.

## 1. Introduction

**Scheme 1:**
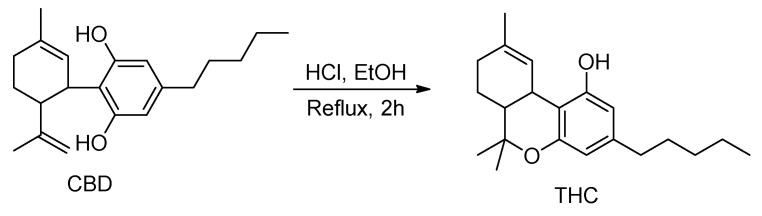
Conversion of CBD to THC [1,2,3].

Cannabidiol (CBD) is a non-psychoactive cannabinoid extracted from *Cannabis sativa* [1,2,3], which can be easily transformed to psychoactive ∆^9^-tetrahydrocannabinol (THC) (Scheme 1), making CBD, a banned chemical in many countries [4].

In addition to its anticonvulsant activity, CBD is also famous for its antimicrobial and antioxidant activities [5,6,7], with potential therapeutic effects on cutaneous conditions [8,9,10]; it has thereby been used in cosmetics and for other antimicrobial applications [9,11,12]. Meanwhile, in addition to CBD, CBD analogues are also expected to form a new class of antibiotics [13,14,15], but research on their structure–function relationship is still lacking. 

Hence, many efforts have been made to discover CBD alternatives either through plant extraction or synthetic approaches. Since plant extraction is costly and time consuming [16] due to the enormous cannabinoid levels in the plant [17,18,19], the synthetic approach has come a long way, from the hydrogenation of CBD [20] to synthesis without the use of CBD as a starting material [21,22]. 

**Scheme 2:**
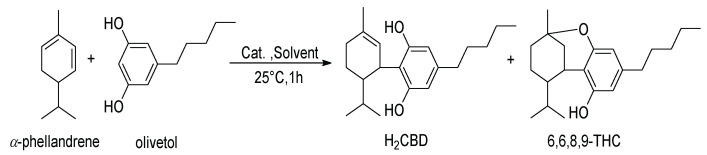
Synthetic route of H_2_CBD and 6,6,8,9-THC [22].

The hydrogenation of CBD results in two products [20], including 8,9-dihydrocannabidiol (H_2_CBD), which was found to be non-intoxicating and demonstrates a similar anticonvulsant effect on rats as CBD [21]. Not derived from CBD, H_2_CBD was first synthesized from α-phellandrene and olivetol in 1988 [22] (Scheme 2). Recently, Mascal and coworkers optimized the reaction to increase the H_2_CBD yield from 26% [22] to 71% with *p*-toluenesulfonic acid monohydrate [21]. 

However, the reported synthesis of H_2_CBD still consumes a huge amount of catalyst with low catalytic specificity [21]. Meanwhile, if H_2_CBD is expected to become an alternative to CBD in cosmetics, its skin toxicity and other bioactive properties must be better understood. The properties of the byproduct 6,6,8,9-THC also need to be elucidated.

Therefore, to examine the prospect of H_2_CBD as a CBD alternative in cosmetics, it is necessary to investigate whether H_2_CBD possesses other biological properties such as antioxidant activity, bactericidal activity, synergistic or antagonistic effects with popular antibiotics, and skin toxicity, as CBD does. Meanwhile, more information about the structure–performance of CBD analogues is needed in order to develop synthetic molecules and determine their properties. 

**Scheme 3:**
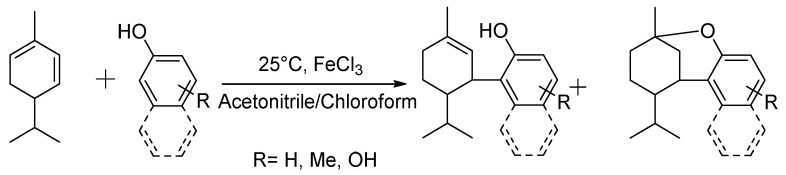
Synthesis of other cannabinoid analogues (this work).

Herein, to examine the structure–performance characteristics of CBD analogues in their antimicrobial and antioxidant aspects, and to verify whether the CBD analogues, and H_2_CBD in particular, could act as CBD alternatives, H_2_CBD (Scheme 2) and seven other CBD analogues (Scheme 3) were synthesized, and their antibacterial and bactericidal activity was studied, with a comparison between CBD and four popular antibiotics; the skin toxicity towards human skin fibroblasts was also evaluated. A possible synergistic effect between H_2_CBD and the antibiotics was also investigated. The antioxidant activity of CBD and the CBD analogues was compared with vitamin C, in terms of radical scavenging activity and inhibition on lipid oxidation, as well as the thermostability of their antioxidant activity.

## 2. Results

### 2.1. Synthesis of CBD Analogues and Improvement on H_2_CBD Production

The reaction between olivetol and α-phellandrene is a typical Friedel–Crafts alkylation, which can be catalyzed by Lewis acid in solvent, resulting a diastereoisomer and accompanied by an intramolecular electrophilic addition as a by-reaction [22]. The reported synthesis of H_2_CBD still requires a huge amount of catalyst (*p*-toluenesulfonic acid monohydrate, 30% eq of the starting material) [21], obstructing the scale-up of production. To improve the synthesis of H_2_CBD based on previous studies [21,22], as previously mentioned, the catalysts and solvents were screened based on their reaction specificity and their production yield of H_2_CBD (Table 1) first.

As presented in Table 1, FeCl_3_ (Run 1) exhibited slightly better catalytic activity than AlCl_3_ (Run 6), and presented the best catalysis in chloroform (Run 3–5). The production yields of H_2_CBD in different solvents corresponded to the polarity sequence of the solvents (Run 1–3): chloroform > benzene > toluene. With 5%–10% (eq.) of FeCl_3_, a production yield of H_2_CBD of 80.7–85.2% was achieved with almost 100% selectivity (Run 4–5). Therefore, FeCl_3_ was chosen as the catalyst for the preparation of the remaining cannabinoid analogues.

Subsequently, the remaining cannabinoid analogues were prepared for the analysis of their structure–function relationships. The production yields of the other cannabinoid analogues are presented in Table 2, wherein the reactions were conducted with α-phellandrene and other phenols or naphthols under the catalysis of FeCl_3_ in chloroform and acetonitrile (1:1, *v*/*v*). 

In Table 2, Compounds **1b**, **2b**, and **3b** were produced from the intramolecular electrophile addition of Compounds **1a**, **2a**, and **3a**, respectively. The yield of the electrophile addition depends on the hydrogen withdrawal from the hydroxyl and the steric hindrance, which is concordant with the structural features of the carbocations. 

The NMR and MS profiles (Figures S1–S9) and the data of the as-prepared CBD analogues including H_2_CBD are provided in the supporting material. 

Hence, the as-prepared CBD analogues are used for the following tests on antibacterial and antioxidant activities.

### 2.2. H_2_BCD and CBD Present Similar and Stronger Antibacterial and Bactericidal Activity Than Popular Antibiotics

**Figure 1:**
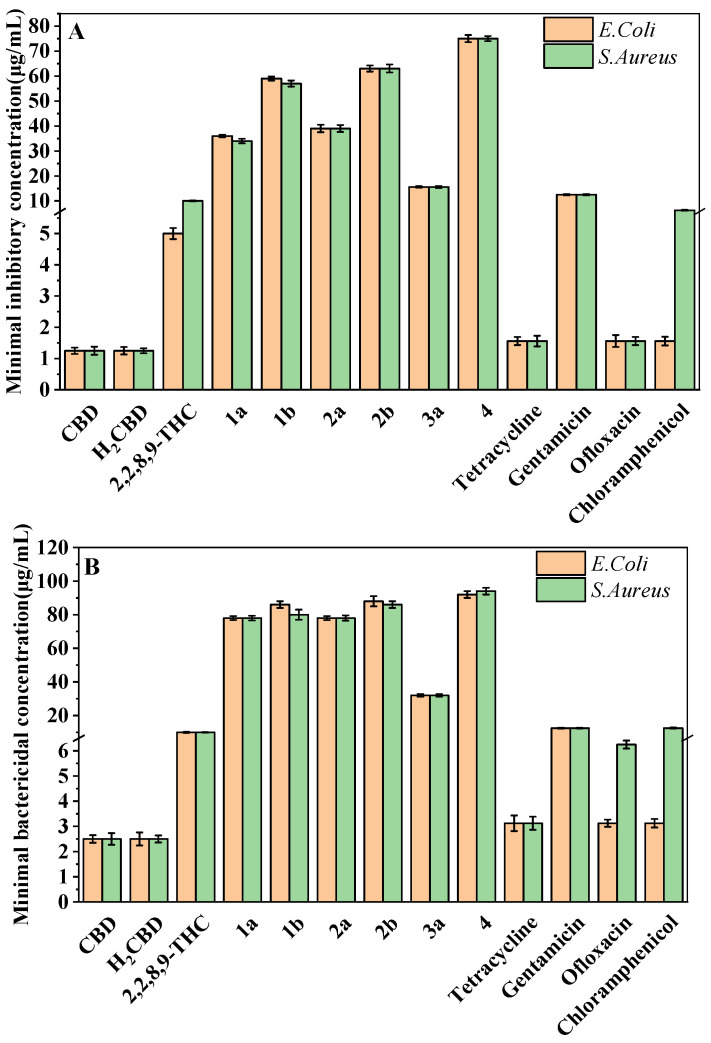
MIC (**A**) and MBC (**B**) of H_2_CBD, CBD, 6,6,8,9-THC, Compounds **1a**, **1b**, **2a**, **2b**, and **3a**, and the antibiotics against *S. aureus* and *E. coli*.

The most attractive or characteristic properties for the use of CBD in cosmetics are antibacterial activity and antioxidant activity. Hence, the antibacterial and bactericidal activity of H_2_CBD was evaluated against *S. aureus* and *E. coli*, with the comparison of CBD, 6,6,8,9-THC, and other CBD analogues, as well as four popular antibiotics (tetracycline hydrochloride, gentamicin, ofloxacin and chloramphenicol), presented as the minimum inhibitory concentration (MIC) and minimum bactericidal concentration (MBC), respectively (Figure 1). 

Figure 1 indicates that both CBD and H_2_CBD possess very similar and much stronger antibacterial activity than the popular commercial antibiotics, and both presented the lowest MICs and MBCs in the assayed samples. The MIC of CBD was 1.25 ± 0.10 μg/mL to *E. coli* and 1.25 ± 0.13 μg/mL to *S. aureus*, whereas the MIC of H_2_CBD was 1.25 ± 0.12 μg/mL to *E. coli* and 1.25 ± 0.08 μg/mL to *S. aureus*. 

In addition to H_2_CBD, 6,6,8,9-THC also demonstrated stronger antibacterial activity than the other assayed CBD analogues, providing similar MIC/MBC to that of gentamicin and chloramphenicol (Figure 1). All phenols (Compounds **1a**, **2a**, and **3a**) exhibited lower MICs/MBCs than their addition products (Compounds **1b**, **2b**, and **3b**); Compound 3b did not present an obvious antibacterial effect. Compound **4** had the most hydroxyl groups but presented the highest MIC/MBC, which implies that the number of hydroxyl groups is not a significant factor in the antibacterial activity of phenolic compounds, which somehow does not fit expectations.

Thus, H_2_CBD may be a potential alternative to CBD in the antimicrobial aspect, and as an antibacterial agent, 6,6,8,9-THC could be employed together with H_2_CBD to save the purification process after the synthesis.

### 2.3. H_2_CBD Does Not Present Synergistic or Antagonistic Effect on Bacteria with the Popular Antibiotics

**Figure 2:**
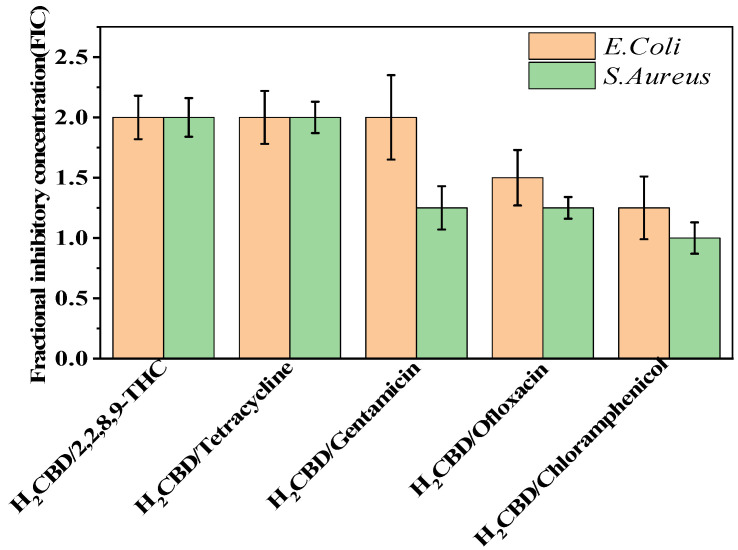
Synergy test on *S. aureus* and *E. coli*.

Based on the superior antibacterial and bactericidal activities of H_2_CBD, we became interested in whether H_2_CBD would present a synergistic or antagonistic effect towards the bacteria with the assayed antibiotics. Hence, the synergistic effects of H_2_CBD and 6,6,8,9-THC with tetracycline hydrochloride, gentamicin, chloramphenicol, and ofloxacin against *S. aureus* were verified on the antibiotic resistance of drug combinations. However, Figure 2 presents the findings that all the FIC indexes were in the range of 1–2, indicating that there was no obvious synergistic or antagonistic effect between H_2_CBD and its analogues or other antibiotics. 

Therefore, H_2_CBD and CBD may serve as alternatives to tetracycline hydrochloride, gentamicin, chloramphenicol, and ofloxacin against *S. aureus*, making them synthetic or botanic antibiotics, respectively.

### 2.4. H_2_CBD Follows CBD in the Time–Kill Kinetics Curves

**Figure 3:**
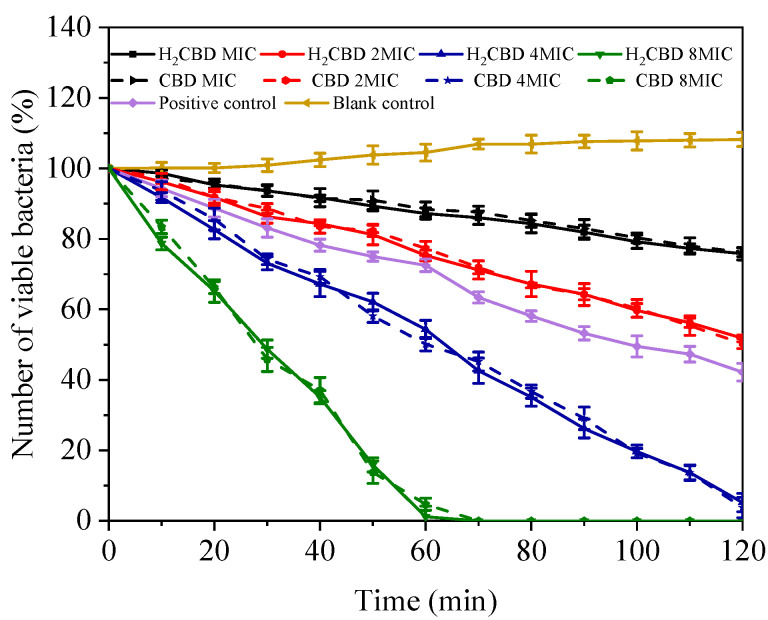
Time–kill kinetics curves of *S. aureus* under treatment of minimum inhibitory concentration (MIC), 2 × MIC, 4 × MIC, and 8 × MIC of H_2_CBD, CBD.

To compare the kinetic bactericidal effect of H_2_CBD and CBD, a time–kill assay towards the growth of *S. aureus* was conducted over time in the presence of MIC, 2 × MIC, 4 × MIC, and 8 × MIC of H_2_CBD and CBD (Figure 3); tetracycline (at the concentration of its MIC) was set as the positive control. 

As Figure 3 indicates, at all assayed concentrations and times, the performance of H_2_CBD mirrored that of CBD; in another words, H_2_CBD possessed similar kinetic bactericidal effect to CBD. In addition, after 24 h, the plates inoculated with the above drug-containing bacteria liquid showed no colonies, further confirming the bactericidal effect of H_2_CBD and its potential as an alternative to CBD in respect to its bactericidal properties. 

### 2.5. H_2_CBD Possess Similar Toxicity towards to Human Skin Fibroblasts as CBD

**Figure 4:**
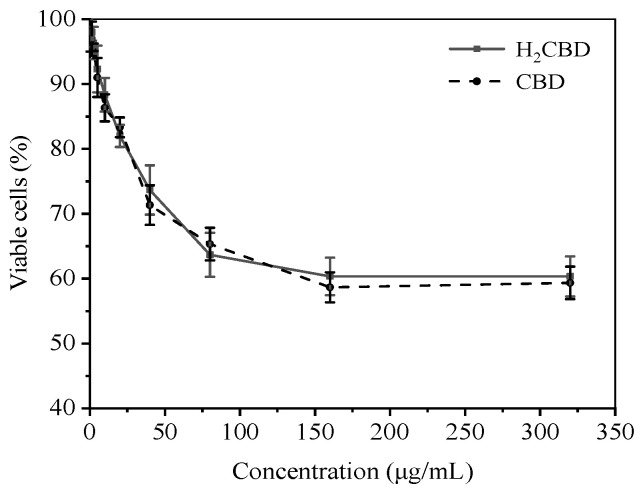
Toxicity of H_2_CBD or CBD towards human skin fibroblasts.

H_2_CBD presented similar antibacterial effects to CBD, yet its application in cosmetics also raises concerns regarding its toxicity towards human skin. So, the safety of H_2_CBD on human skin fibroblasts was evaluated with MTT assay, including a comparison with CBD (Figure 4). The cells were treated with 1.25–320 μg/mL of H_2_CBD and CBD for 24 h, respectively; and the percentage of cell survival was analyzed. 

Figure 4 indicates that the H_2_CBD showed a dose-dependent toxicity in the range of 1.25 μg/mL to 80 μg/mL, and the viable cells stabilized at around 60% at a H_2_CBD concentration of up to 320 μg/mL. Both CBD and H_2_CBD presented lower toxicity to human skin fibroblasts at concentrations up to 64-fold higher than their MIC values (1.25 μg/mL) against *S. aureus*.

In view of the similar antibacterial and bactericidal effects, as well as the toxicity of CBD and H_2_CBD towards human skin fibroblasts, H_2_CBD appears to be a promising alternative to CBD in the relative fields. Next, we present the analysis of the antioxidant activity of these CBD analogues.

### 2.6. H_2_CBD Possesses Similar Antioxidant Activity to CBD and Is Comparable with VC

**Figure 5:**
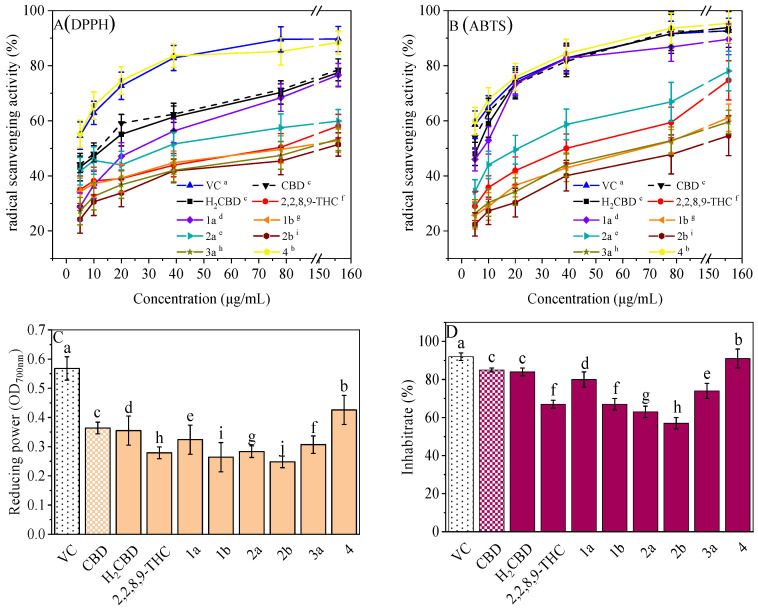
Antioxidant activities of the H_2_CBD with other antioxidants. DPPH (**A**), ABTS (**B**), FRAP (**C**), Lipid oxidation inhibition (**D**). Data are expressed as mean SD (n = 3). Different letters indicate statistically significant difference according to Duncan’s test at the significance level (*p* < 0.05).

Antioxidant activity, especially radical scavenging ability and inhibition on lipid oxidation, plays an important role in cosmetics. Most phenolic compounds demonstrate antioxidant activity, so do CBD and its phenolic analogues (Figure 5). They all exhibited concentration-dependent antioxidant activity with respect to the radical scavenging ability (Figure 5A,B), antioxidant capacity for the reduction of ferric ions (Figure 5C), and the inhibition of lipid oxidation (Figure 5D).

It is interesting that Compound **4** exhibited the best antioxidant activity of all the assays (Figure 5), although it presented the weakest antibacterial effect (Figure 1). The antioxidant activity of Compound **4** was even close to that of VC. Compound **3b** also did not present activity in all antioxidant assays. This may imply that a hydroxyl moiety is an essential group for CBD analogues to carry out their antibacterial and antioxidant activities. 

For the other analogues, no obvious performance–structure relationship was observed. Again, H_2_BCD possessed similar antioxidant activity to that of CBD, although their activity was weaker than that of Compound **4**. 

**Figure 6:**
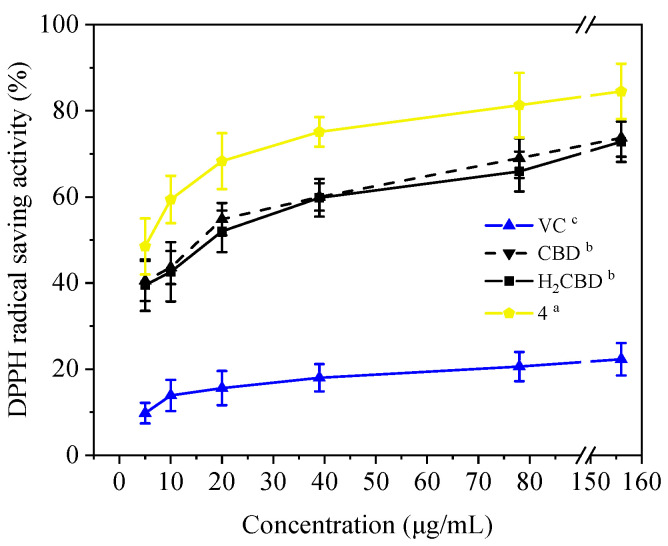
DPPH assay of the CBD analogues and VC at 80 °C. Different letters present the data significance.

Moreover, in addition to demonstrating antioxidant activity at room temperature, these three (H_2_BCD, CBD, and Compound **4**) demonstrated higher and more stable antioxidant activity at 80 °C, overtaking VC (Figure 6).

Therefore, H_2_BCD and Compound **4**, which have the most phenolic hydroxyl groups, may be alternatives to CBD in terms of antioxidant activity.

## 3. Discussion

### 3.1. Synthesis of H_2_CBD

H_2_CBD can be obtained together with H_4_CBD from the hydrogenation of CBD [13,20]. Without CBD as the starting material, Mascal et al. prepared H_2_CBD with olivetol and α-phellandrene under the catalysis of *p*-toluenesulfonic acid monohydrate (30% eq.), resulting in a yield of H_2_CBD of 71% [21], which was optimized based on what Crombie reported. The authors did not mention the catalytic selectivity or the yield of 6,6,8,9-THC [21]. 

In general, the best Lewis acid catalyst for a regular Friedel–Crafts reaction would be AlCl_3_. As presented in Table 1, FeCl_3_ (Run 1) demonstrated slightly better catalytic activity than AlCl_3_ (Run 6). Considering the cost and safety, FeCl_3_ was selected for the subsequent experiments (Run 1–5). 

Table 1 indicates that the catalytic activity of FeCl_3_ is dramatically influenced by the solvent polarity, which can be explained by the fact that a solvent with a higher polarity would better stabilize the formed electrophilic carbocation generated from olivetol. Hence, FeCl_3_ presented the best catalysis in chloroform (Run 3–5), and the production yields of H_2_CBD in different solvents followed the polarity sequence (Run 1–3): chloroform > benzene > toluene. With 5–10% (eq.) of FeCl_3_, a production yield of H_2_CBD of 80.7–85.2% was achieved with nearly 100% selectivity (Run 4–5). 

In addition to the solvent, the intramolecular addition reactivity also varied dramatically depending on the catalyst (Table 1), resulting in remarkable differences in the reaction selectivity. The intramolecular addition of CBD may be driven by acidic conditions that are responsible for further electrophile additions toward the more thermodynamically stable compounds [23]. This may explain why the higher catalyst dosage would yield more additional byproducts, as seen in Table 1.

In this Friedel–Crafts reaction, diastereoisomer and regioisomer can be formed, so the products obtained in Table 1 and Table 2 are diastereoisomers [13]. As Blaskovich pointed out, the dihydrocannabidiol regioisomers presented in the NMR data resemble each other [13], and the NMR and MS data in the supporting material confirm their structures.

### 3.2. The Antibacterial Activity of H_2_BCD and CBD in Comparison to Popular Antibiotics

Although it is well-known that CBD possess antibacterial properties [6,13,24,25,26,27], its efficacy, according to different groups, varies widely. Blaskovich studied the antibacterial activity of CBD against highly resistant strains of *S. aureus*, and confirmed the gram-positive inhibition of CBD [13]. Martinenghi et al. reported that both CBD and its analogue, cannabidiolic acid (CBDA), showed significant antimicrobial effects against the gram-positive *S. aureus* and *Staphylococcus epidermidis*, but no activity was noticed against gram-negative *E. coli* and *P. aeruginosa* [24]. However, a reduction of 11% (*p* = 0.0161) on *E. coli* cell viability was observed with 5 µm of CBD, but no significant effect was observed on *S. aureus* cell viability, as assessed by a disk diffusion test [25]. 

The results in Figure 1 are differ slightly to those that Martinenghi reported, in which the MIC of CBD is higher than 64 μg/mL to *E.coli* and 1 μg/mL to *S. aureus* [24]. This difference could be due to the strain difference; the *E. coli* (ATCC 25922) they assayed [24] is a recommended reference strain that may be a resistant bacterium, and another study on the performance of CBD against resistant bacteria [28] revealed an MIC value above 128 µg/mL for CBD against *E. coli* (UTI89). 

As a CBD analogue, the antibacterial activity of H_2_CBD has been reported occasionally. It is interesting to note that although Compounds **1a** and **2a** and H_2_CBD are phenols each containing two phenolic hydroxyl groups, their antibacterial activities are starkly different (Figure 1). That might be caused by the only structural difference, the alkyls attached to the benzene ring, together with the phenomena presented by Compound **4**, which might imply that an appropriate amphipathy is needed for antibacterial activity since H_2_CBD has a longer alkyl moiety. Appendino et al. studied the structure–activity of antibacterial cannabinoids obtained from *Cannabis sativa*, and their results suggest that the prenyl moieties of cannabinoids serve mainly as modulators of lipid affinity for the olivetol core; the authors claimed that the high potency of cannabinoids suggests a specific yet elusive mechanism of their activity [28]. They also found that the methylation and acetylation of the phenolic hydroxyls and the introduction of a second prenyl moiety were detrimental for antibacterial activity; this is in accordance with our findings from the alkylation of the phenolic hydroxyl (Compounds **1b**, **2b**, and **3b**). 

As for the synergistic or antagonistic effects of popular antibiotics towards the bacteria, CBD was reported to potentiate the effect of bacitracin (BAC) against gram-positive bacteria (*Staphylococcus species, Listeria monocytogenes,* and *Enterococcus faecalis*) but appears ineffective against gram-negative bacteria; CBD reduced the MIC of BAC at least 64-fold and the combination yielded an FIC index of 0.5 or below in most gram-positive bacteria tested [25]. Furthermore, the synergistic or antagonistic effects of CBD analogues in combination with popular antibiotics towards bacteria remain unclear [25]. Our findings do indicate that the combination is not synergistic; however, it was close to synergistic.

In addition, the performance of CBD in the time–kill kinetics experiment is in accordance with what has been recently reported [24]. The results also show that both H_2_CBD and CBD presented a significant inhibitory effect on the growth of bacterial strains over the time. It is notable that in the time–kill profile the data dots of H_2_CBD and CBD remained close, indicating a very similar kinetic bactericidal performance.

### 3.3. The Toxicity of H_2_BCD and CBD towards Human Skin Fibroblasts 

CBD has demonstrated several beneficial skin properties [11,29,30,31]. CBD reduced reactive oxygen species levels in human dermal fibroblasts better than vitamin C [31]. As H_2_BCD possesses similar low toxicity towards human skin fibroblasts as CBD does, H_2_BCD is a promising option for use in cosmetics, as an alternative to CBD, in terms of the safety inherent with a botanic chemical.

### 3.4. The Antioxidant Activity of H_2_BCD and CBD in Comparison of VC

The antioxidant activity of CBD [32] as well as its combined regimens with other antioxidants [33] have drawn much attention recently The antioxidant and anti-inflammatory effects of CBD in monocrotaline-induced pulmonary hypertension have been reported [34]. CBD was found to be more effective than vitamin C and E as a neuroprotective antioxidant [35] Herein, H_2_CBD and Compound **4** presented similar antioxidant activity as VC, but exhibited a much higher thermostability of antioxidant activity than VC.

## 4. Materials and Methods

### 4.1. Chemicals and Reagents

CBD (98%, HPLC purity) was purchased from Guilin Layn Natural Ingredients Corp., Guangxi, China. Olivetol (98%, HPLC purity) was purchased from Shenghong Corporation, Shanghai, China. α-Phellandrene (85%, HPLC purity) was purchased from Sigma-Aldrich, Shanghai, China. BF_3_·2H_2_O (AR, 99%), CuCl_2_·2H_2_O (AR, 97%), AlCl_3_ (AR, 99%), and FeCl_3_ (AR, 98%) were purchased from Macklin Biochemical Co., Ltd., Shanghai, China. All other chemicals were purchased from Sinopharm Chemical Reagent Co., Ltd., Shanghai, China. All the chemicals were of analytical grade and used as received unless otherwise stated. 

*Escherichia coli* (CICC 20549) and *Staphylococcus aureus* (CICC 23656) strains were supplied by the China Center of Industrial Culture Collection (CICC), Beijing, China. Tetracycline hydrochloride, gentamicin, chloramphenicol, and ofloxacin were obtained from Sinopharm Chemical Reagent Co., Ltd., Shanghai, China.

### 4.2. Preparation of 8,9-Dihydrocannabidiol (H_2_CBD) and NMR Profiles of CBD Analogues

In a typical procedure, 0.081 g of FeCl_3_ (0.05 mmol) was suspended in the solution of olivetol (1.8 g, 10.0 mmol) and α-phellandrene (1.5 g, 11 mmol) in chloroform (10 mL). The suspension was then stirred at 25 °C for 1 h. The reaction was monitored with TLC (petroleum:ethyl acetate = 40:1, *v*/*v*). Next, the reaction was completed and the solvent was removed from the reaction mixture under vacuum. The resultant residue was separated with gradient column chromatography, and eluted with the mixture of petroleum and ethyl acetate (100:1 to 40:1, *v*/*v*) to obtain H_2_CBD (2.7 g, production yield of 85%) as a yellow oil. The byproduct was confirmed as 6,6,8,9-tetrahydrocannabinol (6,6,8,9-THC) with the analysis of NMR and MS.

^1^H and ^13^C NMR spectra were recorded on a Bruker AV-400 spectrometer at 400 and 100 MHz, respectively, in CDCl_3_ unless otherwise mentioned. Coupling constants (J) are expressed in hertz (Hz). The chemical shifts (δ) of NMR are reported in parts per million (ppm) units relative to the solvent. EIMS was recorded on a Pegasus BT EI mass spectrometer. HRMS was recorded on a MALDI SYNAPT MS spectrometer.

### 4.3. Minimum Inhibitory Concentration (MIC) and Minimum Bactericidal Concentration (MBC) Assays against S. aureus and E. coli 

The antibacterial activity against *Staphylococcus aureus* and *Escherichia coli* was tested according to the literature with slight modifications [36]. The samples were dissolved in 95% ethanol first to make a stock solution, and then diluted with Luria–Bertani broth to the concentration of 1 mg/mL, followed by the dilutions needed and tested against *S. aureus* and *E. coli*, respectively. All strains were cultivated in Luria–Bertani broth at 37 °C. The bacterial inoculum (100 µL, 10^6^ CFU/mL) was added to wells, and the plate was incubated at 37 °C for 24 h. The MIC was determined as the lowest concentration at which no growth was observed. The drug-containing bacteria liquid was inoculated in the corresponding culture medium plate and continued to be cultured at 37 °C for 24 h. The drug concentration with the minimum dilution of less than five colonies on average was recorded as the minimum bactericidal concentration (MBC). A total of 1% ethanol in Luria–Bertani broth was set as the blank. All data were determined in triplicate and represented as the mean ± SD.

### 4.4. Synergistic Effect of CBD Analogues with the Classic Antibiotics against S. aureus and E. coli

The fractional inhibitory concentration (FIC) index was applied to evaluate the synergistic effect [37] of the CBD analogues with the classic antibiotics against *S. aureus* and *E. coli*. For this test, 72-fold dilutions with LB from 8 MIC to 1/8 MIC of the H_2_CBD, 6,6,8,9-THC, and the four antibiotics (tetracycline hydrochloride, gentamicin, chloramphenicol, and ofloxacin) were employed, respectively. The FIC of the compound was calculated as the ratio of the MIC of the compounds in the combination with the MIC of the compound alone. The FIC index was calculated as the sum of the FIC of the antibiotic (a) and the FIC of H_2_CBD (b).

$$FIC_{a}=\frac{MIC_{a+b}}{MIC_{a}}$$

$$FIC_{b}=\frac{MIC_{b+a}}{MIC_{b}}$$

(1) $$FIC_{index}=FIC_{a}+FIC_{b}$$

The synergistic effect based on the FIC index is defined as follows: an FIC index ≤ 0.5, indicates a synergistic effect; an FIC index 0.5–1 indicates an additive effect; an FIC index 1–4 indicates indifference; an FIC index > 4 indicates antagonism.

### 4.5. Optical Density Measurements for Kinetic Bactericidal Effect of H_2_CBD

The kinetic bactericidal effect of H_2_CBD was determined with the optical density measurement, presented as the time–kill kinetics assay according to the literature [24], with the comparison of CBD. The optical density measurement of the drug-containing bacteria (*S. aureus*) liquid was conducted with a 96-well microtiter plate. H_2_CBD with concentrations of 8 × MIC, 4 × MIC, 2 × MIC, and MIC were added to the wells with 50 μL of bacterial culture (1 × 10^6^ CFU/mL), respectively. The absorbance was measured at 600 nm and every 10 min at 37 °C in 2 h. Sterile control and positive control (1 × MIC of tetracycline) were used in the experiment, and three replicates were set, respectively. After determination, the above drug-containing bacteria liquid was inoculated into the corresponding culture medium plate and continued to be cultured at 37 °C for another 24 h to test the bactericidal effect of H_2_CBD. 

### 4.6. Cytotoxicity of H_2_CBD to Human Skin Fibroblasts

Human skin fibroblasts were inoculated into 96-well plates at a density of 2000/100 μL (three parallels were set in each group) and cultured at 37 °C in 5% CO_2_ for 24 h to allow the cells to adhere completely to the walls [29]. The medium was removed, then 200 μL each of H_2_CBD of different concentrations (dissolved in fresh medium) were added to the wells in the plate, and culturing was continued for 12 h (time can be adjusted according to the specific situation). Each well was pretreated with MTT (5 mg/mL, 20 μL). After incubation for 4 h, the supernatant in the well was carefully absorbed and 150 μL DMSO was added to each well, and the purple crystals were dissolved by vibration for 10 min. The absorbance value of each hole at the wavelength of 490 nm was recorded with a microplate analyzer, and calculated according to the formula:

(2) $$\mathrm{Cell}\;\mathrm{survival}\;\mathrm{rate}\%\;=\frac{\;\mathrm{absorbance}\;\mathrm{of}\;\mathrm{the}\;\mathrm{sample}}{\begin{array}{c} \mathrm{absorbance}\;\mathrm{of}\;\mathrm{the}\;\mathrm{blank} \end{array}}\times 100\%$$

### 4.7. DPPH Radical Scavenging Activity

The DPPH radical scavenging activity was measured according to the literature [38], with some modifications. Briefly, 50 μL of DPPH ethanol solution (0.06 mmol/L) and 50 μL of sample ethanol solution were mixed in a well of a 96-well microtiter plate and incubated at testing temperature for 30 min in dark. The absorbance of the reaction mixture was measured at 517 nm. Ethanol alone in the reaction mixture served as the control. A sample blank was prepared by replacing the DPPH with ethanol. The DPPH radical scavenging activity was calculated according to the following equation:

(3) $$\mathrm{DPPH}\;\mathrm{inhibition}\;\%\;=\frac{A_{\mathrm{control}}-\left(A_{\mathrm{sample}}-{\;A}_{\mathrm{sample}\;\mathrm{blank}}\right)}{A_{\mathrm{control}}}\times 100\%$$

The DPPH radical scavenging capacity of the samples was plotted against the sample concentrations to determine the IC50 values.

### 4.8. ABTS Radical Scavenging Assay

The ABTS radical scavenging activity was examined according to the literature [38]. The ABTS solution containing ABTS (final concentration of 7 mmol/L) and potassium persulphate (final concentration of 2.45 mmol/L) was incubated in the dark at room temperature for 16 h. The mixture was adjusted with ethanol to obtain an absorbance of 0.700 ± 0.02 at 734 nm. The diluted ABTS solution (50 μL) was mixed with 50 μL of sample in each well on a 96-well microtiter plate and stored at room temperature for 10 min. Then, the absorbance of the mixture was recorded at 734 nm. Ethanol alone in the reaction mixture served as control. A sample blank was prepared by replacing the ABTS with ethanol. The ABTS radical scavenging activity is expressed as ABTS (%) and calculated as below:

(4) $$\mathrm{ABTS}\;\mathrm{inhibition}\;\%\;=\frac{A_{\mathrm{control}}-\left(A_{\mathrm{sample}}-{\;A}_{\mathrm{sample}\;\mathrm{blank}}\right)}{\begin{array}{c} A_{\mathrm{control}} \end{array}}\times 100$$

The radical scavenging ability of ABTS was plotted against the sample concentrations to determine the IC50 values.

### 4.9. Ferric Reducing Antioxidant Power Assay

A ferric reducing power was tested using a modified FRAP assay [39]. The sample solutions were mixed with 2.5 mL of phosphate buffer (0.2 M, pH 6.6) and 2.5 mL of potassium ferricyanide (1%). After incubating the reaction mixture for 20 min at 50 °C, 2.5 mL of trichloroacetic acid (10%) was added to each test tube, and the mixture was centrifuged at 8000 rpm for 10 min. The supernatant (2.5 mL) was mixed with 2.5 mL of distilled water and 0.5 mL of ferric chloride (0.1%). The absorbance of the reaction mixture was recorded at 700 nm. 

### 4.10. Lipid Oxidation Inhibition Assay

The thiobarbituric acid reactive substances (TBARS) assay was conducted using the modified method of Murzakhmetova [40] as a measurement of the lipid oxidation. Briefly, 0.15 mL of hydrogen peroxide (2 mg/mL) was mixed with 0.5 mL of olive oil ethanol solution (mass ratio, 1:3) in a test tube, then 1 mL of sample ethanol solution and 2 mL of 2-thiobarbituric acid ethanol solution (0.2 wt%) were pipetted into the tube, respectively, and incubated at 37 °C for 25 min. After that, the mixture was incubated at 90 °C for 30 min with 2 mL of trichloroacetic acid (20%) and then cooled to room temperature. Afterwards, 1 mL of trichloromethane was employed to extract the organic phase, and the absorbance of the supernatant was determined at 532 nm for the calculation of lipid oxidation inhibition. The control sample was prepared by replacing the hydrogen peroxide with ethanol. The inhibition rate of lipid oxidation was calculated as following:

(5) $$\mathrm{Lipid}\;\mathrm{oxidation}\;\mathrm{inhibition}\;\%\;=\frac{A_{\mathrm{control}}-\left(A_{\mathrm{sample}}-{\;A}_{\mathrm{blank}}\right)}{A_{\mathrm{control}}}\times 100\%$$

### 4.11. Statistics

All data obtained in this study were analyzed using IBM SPSS Statistics (version 26; SPSS Inc., Chicago, IL, USA). The results are presented as the mean ± standard deviation (n = 3). *p* < 0.05 was considered significant (Duncan’s test).

## 5. Conclusions

In this experiment, several CBD analogues were synthesized with a one pot Friedel–Crafts alkylation with phenols and α-phellandrene, catalyzed by Lewis acid in solvent. FeCl_3_ performed best catalytic activity, and its catalytic activity dramatically depends on the solvent polarity. Typically, the production yields of 8,9-dihydrocannabidiol (H_2_CBD) followed the sequence of solvent polarity: chloroform > benzene > toluene. A nearly 100% of catalytic selectivity towards H_2_CBD was achieved with a production yield of 85% in 1 h at room temperature.

Among the synthesized CBD analogues, H_2_CBD performed much stronger in terms of inhibitory activity against the bacteria than the four assayed popular antibiotics (tetracycline hydrochloride, gentamicin, chloramphenicol, and ofloxacin). The MIC of CBD is 1.25 ± 0.10 μg/mL to *E. coli* and 1.25 ± 0.13 μg/mL to *S. aureus*, whereas the MIC of H_2_CBD is 1.25 ± 0.12 μg/mL to *E. coli* and 1.25 ± 0.08 μg/mL to *S. aureus*. The addition product of H_2_CBD, that is, 6,6,8,9-THC, also demonstrated stronger antibacterial activity than the other CBD analogues did, performing similar MIC/MBC as that of gentamicin and chloramphenicol. H_2_CBD does not present synergistic or antagonistic effects with the popular antibiotics towards the bacteria. 

The structure–antibacterial effect study shows that an appropriate amphipathy might be necessary for antibacterial activity. Compound **4** had the most hydroxyl groups but presented the highest MIC/MBC and antioxidant activity, which implies that the number of hydroxyl groups is not an essential aspect of antibacterial activity in CBD analogues, but may benefit to their antioxidant activity. All CBD analogues exhibited lower MICs/MBCs than their addition products. It was found that an appropriate amphipathy might be needed for antibacterial activity since H_2_CBD has a longer alkyl moiety. The number of phenolic hydroxyls might not be the essential for antibacterial activity but may be beneficial for antioxidant activity. All of the obtained CBD analogues exhibited lower MICs/MBCs than their intramolecular addition products.

In comparison to CBD, H_2_CBD presented extremely similar antibacterial activity against *E. coli* and *S. aureus*, similar antioxidant activity, as well as lower toxicity to human skin fibroblasts. Time–kill assay also confirmed the similar bactericidal effect of H_2_CBD and CBD. H_2_BCD, CBD, and Compound **4** performed higher and more stable antioxidant activity at 80 °C, whereas VC was denatured under the same conditions. 

Taken together, H_2_CBD may be a potential alternative to CBD in terms of antimicrobial and antioxidant action, and could be a promising functional agent for the production of cosmetics.

## Figures and Tables

**Table 1:** Reaction condition screening for H_2_CBD production.

Run	Catalyst	Catalyst: Olivetol (mol %)	Solvent	H_2_CBD Production Yield (%)	6,6,8,9-THC Production Yield (%)
1	FeCl_3_	30	benzene	73.4	10.5
2	FeCl_3_	30	toluene	70.0	12.5
3	FeCl_3_	30	chloroform	85.3	Trace
4	FeCl_3_	10	chloroform	85.2	Trace
5	FeCl_3_	5	chloroform	80.7	Trace
6	AlCl_3_	30	benzene	69.2	12.6
7	BF_3_·H_2_O	30	benzene	48.2	8.7
8	CuCl_2_·2H_2_O	30	benzene	32.7	Trace
9	TsOH	30	benzene	70.5	14.7

**Table 2:** Production yields of the synthetic cannabinoid analogues other than H_2_CBD.

Entry	Phenol/ Naphthol	Product	Compound A Yield (%)	Compound B Yield (%)
1	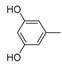	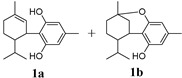	55	25
2	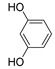	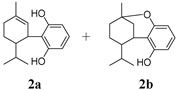	44	30
3	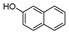	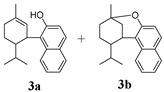	65	20
4	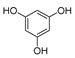	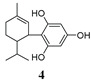	72	/

## Data Availability

Not applicable.

## Supplementary Materials

The following supporting information can be downloaded at: https://www.mdpi.com/article/10.3390/molecules28010445/s1, Figure S1. ^1^H NMR(400 MHz, CDCl_3_) and ^13^C NMR(126 MHz, CDCl_3_) and MS spectra of 8,9-dihydrocannabidiol (H_2_CBD); Figure S2: ^1^H NMR(400 MHz, CDCl_3_) and ^13^C NMR(126 MHz, CDCl_3_) and MS spectra of 6,6,8,9-tetrahydrocannabinol (6,6,8,9-THC); Figure S3: ^1^H NMR(400 MHz, CDCl_3_) and ^13^C NMR(126 MHz, CDCl_3_) and MS spectra of 2′-Isopropyl-4,5′-dimethyl-1′,2′-dihydro-3′,4′-tetrahydro-[1,1′-biphenyl]-2,6-diol(**1a**); Figure S4: ^1^H NMR(400 MHz, CDCl_3_) and ^13^C NMR(126 MHz, CDCl_3_) and MS spectra of 2′-Isopropyl-4,5′-dimethyl-1′,2′-dihydro-3′,4′,6′-hexahydro-1′,2-methoxybenzo[b]oxacyclooctatrien-6-ol (**1b**); Figure S5: ^1^H NMR(400 MHz, CDCl_3_) and ^13^C NMR(126 MHz, CDCl_3_) and MS spectra of 2′-Isopropyl-5′-methyl-1′,2′-dihydro-3′,4′-tetrahydro-[1,1′-biphenyl]-2,6-diol (**2a**); Figure S6: ^1^H NMR(400 MHz, CDCl_3_) and ^13^C NMR(126 MHz, CDCl_3_) and MS spectra of of 2′-Isopropyl-5′-methyl-1′,2′-dihydro-3′,4′,6′-hexahydro-1′,2-methoxybenzo[b]oxacyclooctatrien-6-ol (**2b**); Figure S7: ^1^H NMR(400 MHz, CDCl_3_) and ^13^C NMR(126 MHz, CDCl_3_) and MS spectra of 2′-Isopropyl-5′-methyl-1′,2′-dihydro-3′,4′-tetrahydrocyclohexyl-6-en-1-yl)naphthalene-2-ol (**3a**); Figure S8: ^1^H NMR(400 MHz, CDCl_3_) and ^13^C NMR(126 MHz, CDCl_3_) and MS spectra of 2′-Isopropyl-5′-methyl-1′,2′-dihydro-3′,4′,6′-hexahydro-1,7′-methylnonaphthalene [2,1b] oxoxine (**3b**); Figure S9: ^1^H NMR(400 MHz, CDCl_3_) and ^13^C NMR(126 MHz, CDCl_3_) and MS spectra of 2′-Isopropyl-5′-methyl-1′,2′-dihydro-3′,4′-tetrahydro-[1,1′-biphenyl]-2,4,6-triol (**4**).
