# Supplementary material for: 8,9-Dihydrocannabidiol, an Alternative of Cannabidiol, Its Preparation, Antibacterial and Antioxidant Ability
Source: Molecules. 2023 Jan 3;28(1):445. doi: 10.3390/molecules28010445 (PMC9824641; doi:10.3390/molecules28010445)
Supplement: Supplementary file 1 [file molecules-28-00445-s001.zip › molecules-1985739-supplementary.pdf]

Supporting Materials

8,9-Dihydrocannabidiol, an alternative of cannabidiol, its preparation, antibacterial and antioxidant ability

Qi Wu [1,2], Mao-yue Guo[2], Liang-hua Zou[3], Qi-qi Wang[2] and Yong-mei Xia[1,2*]

[a] *State Key Laboratory of Food Science and Technology, Jiangnan University, Wuxi, Jiangsu 214122, China*

[b] *School of Chemical and Material Engineering, Jiangnan University, Wuxi, Jiangsu 214122, China*

[c] *School of Life Science and Health Engineering, Jiangnan University, Wuxi, Jiangsu 214122, China*

Caption list

Figure S1. ^{1}H NMR(400 MHz, $CDCl_3$) and ^{13}C NMR(126 MHz, $CDCl_3$) and MS spectra of 8,9-dihydrocannabidiol (H_2CBD)

Figure S2. ^{1}H NMR(400 MHz, $CDCl_3$) and ^{13}C NMR(126 MHz, $CDCl_3$) and MS spectra of 6,6,8,9-tetrahydrocannabinol (6,6,8,9-THC)

Figure S3. ^{1}H NMR(400 MHz, $CDCl_3$) and ^{13}C NMR(126 MHz, $CDCl_3$) and MS spectra of 2'-Isopropyl-4,5'-dimethyl-1',2'-dihydro-3',4'-tetrahydro-[1,1'-biphenyl]-2,6-diol (1a)

Figure S4. ^{1}H NMR(400 MHz, $CDCl_3$) and ^{13}C NMR(126 MHz, $CDCl_3$) and MS spectra of 2'-Isopropyl-4,5'-dimethyl-1',2'-dihydro-3',4',6'-hexahydro-1',2-methoxybenzo[b]oxacyclooctatrien-6-ol (1b)

Figure S5. ^{1}H NMR(400 MHz, $CDCl_3$) and ^{13}C NMR(126 MHz, $CDCl_3$) and MS spectra of 2'-Isopropyl-5'-methyl-1',2'-dihydro-3',4'-tetrahydro-[1,1'-biphenyl]-2,6-diol (2a)

Figure S6. ^{1}H NMR(400 MHz, $CDCl_3$) and ^{13}C NMR(126 MHz, $CDCl_3$) and MS spectra of 2'-Isopropyl-5'-methyl-1',2'-dihydro-3',4',6'-hexahydro-1',2-methoxybenzo[b]oxacyclooctatrien-6-ol (2b)

Figure S7. ^{1}H NMR(400 MHz, $CDCl_3$) and ^{13}C NMR(126 MHz, $CDCl_3$) and MS spectra of 2'-Isopropyl-5'-methyl-1',2'-dihydro-3',4'-tetrahydrocyclohexyl-6-en-1-yl)naphthalene-2-ol (3a)

Figure S8. ^{1}H NMR(400 MHz, $CDCl_3$) and ^{13}C NMR(126 MHz, $CDCl_3$) and MS spectra of 2'-Isopropyl-5'-methyl-1',2'-dihydro-3',4',6'-hexahydro-1,7'-methylnonaphthalene[2,1-b]oxoxine (3b)

Figure S9. ^{1}H NMR(400 MHz, $CDCl_3$) and ^{13}C NMR(126 MHz, $CDCl_3$) and MS spectra of 2'-Isopropyl-5'-methyl-1',2'-dihydro-3',4'-tetrahydro-[1,1'-biphenyl]-2,4,6-triol (4)

^{1}H NMR, ^{13}C NMR and MS Data

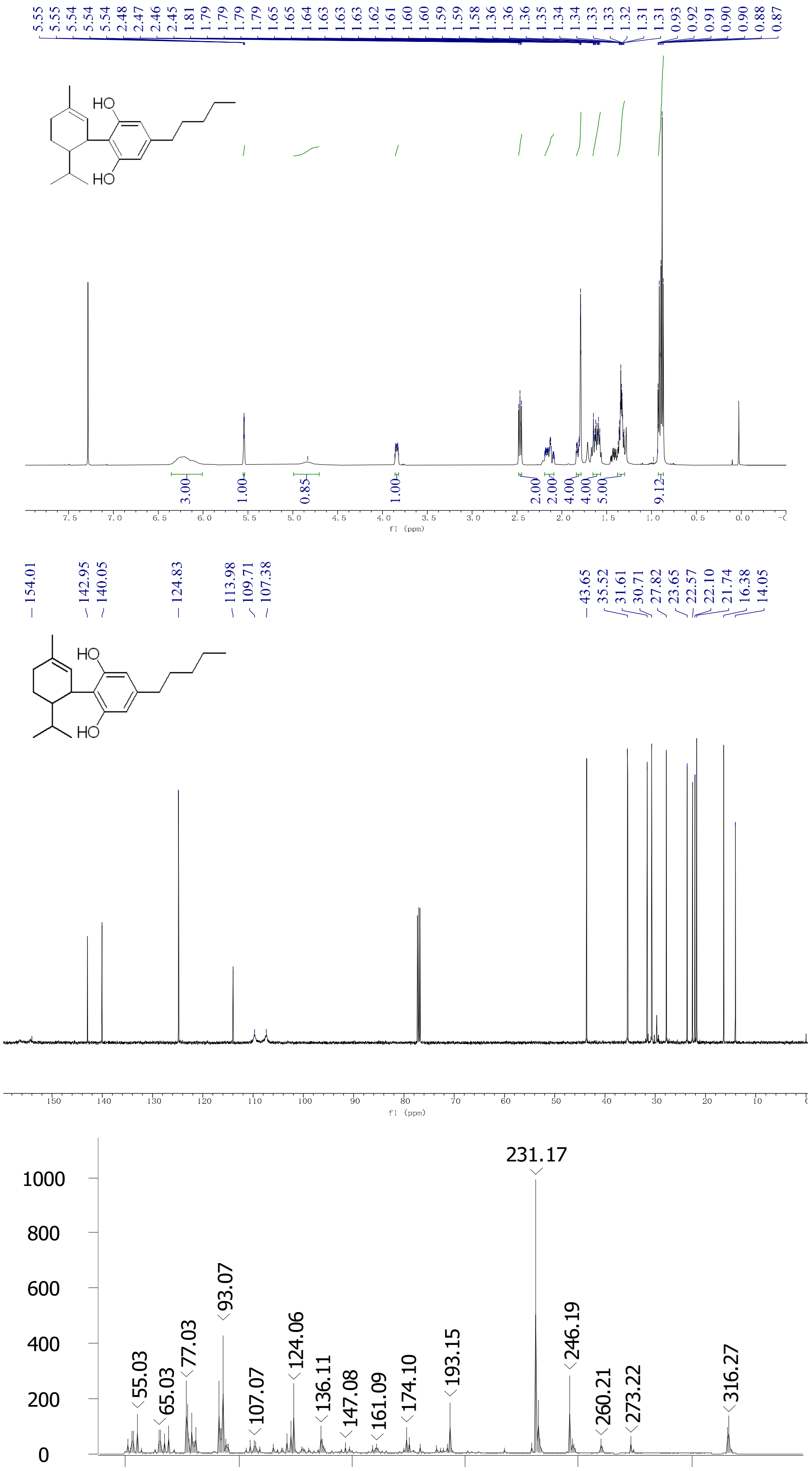

Figure S1. ^{1}H NMR(400 MHz, $CDCl_3$), ^{13}C NMR(126 MHz, $CDCl_3$) and MS spectra of 8,9-dihydrocannabidiol (H$_2$CBD)

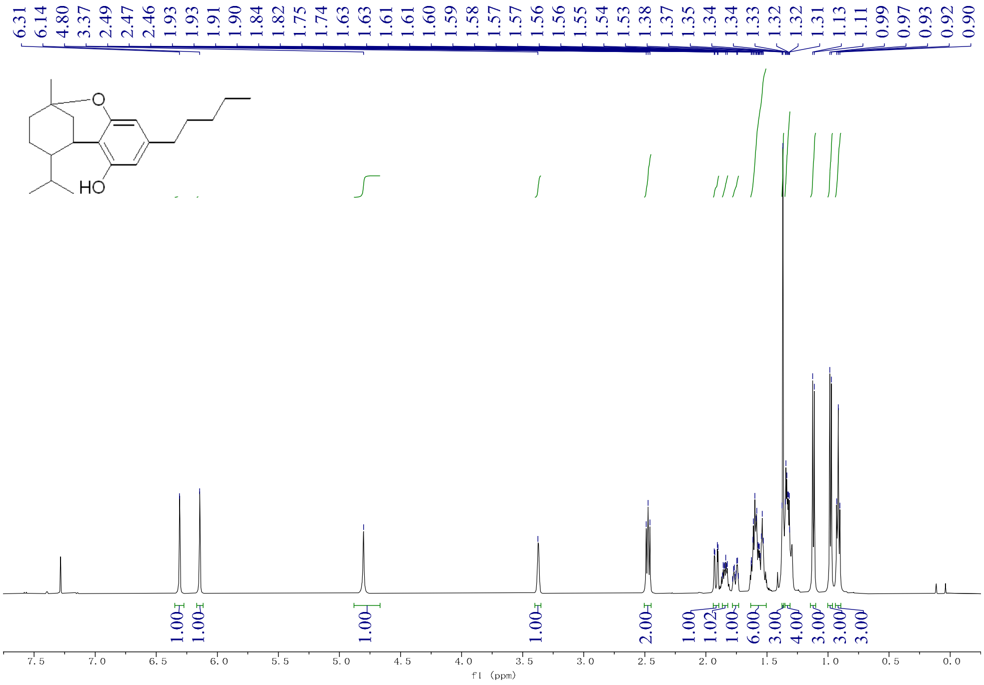

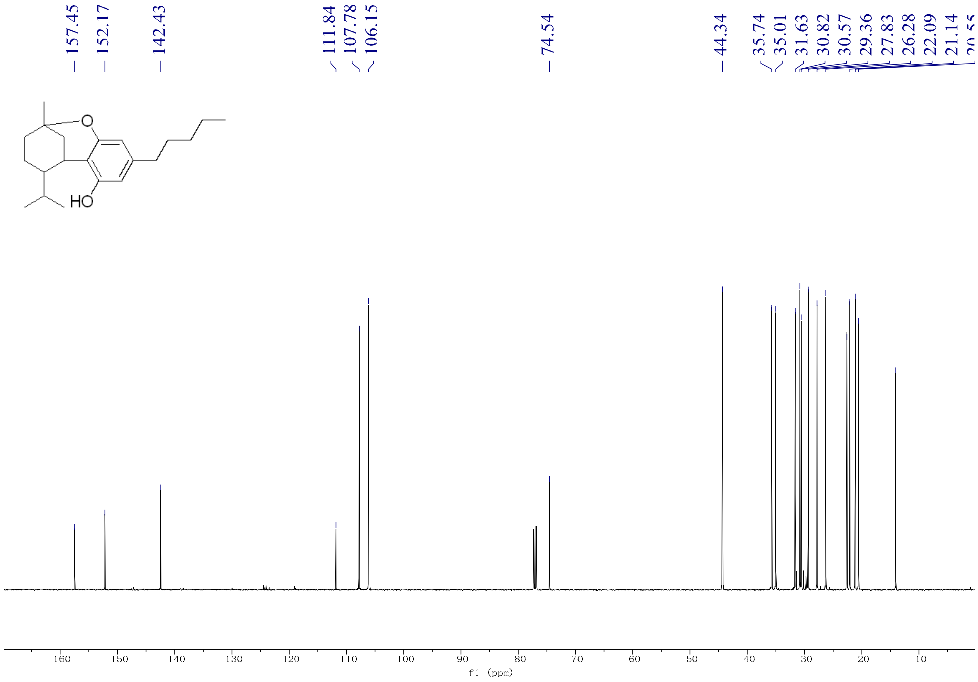

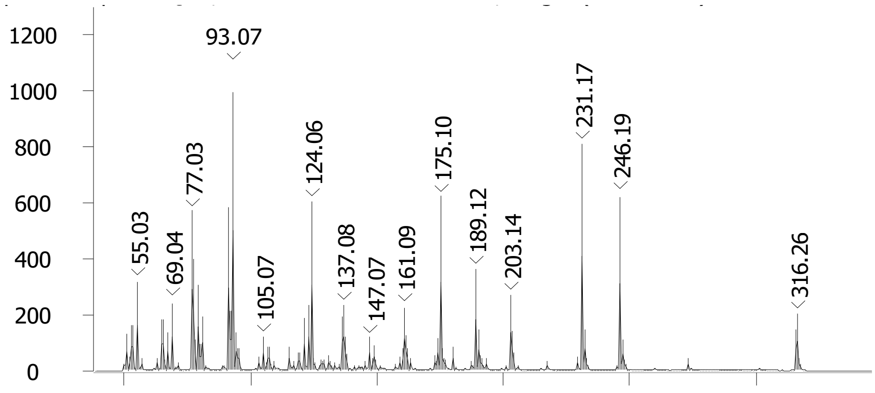

Figure S2. ^{1}H NMR(400 MHz, $CDCl_3$), ^{13}C NMR(126 MHz, $CDCl_3$) and MS spectra of 6,6,8,9-tetrahydrocannabinol (6,6,8,9-THC)

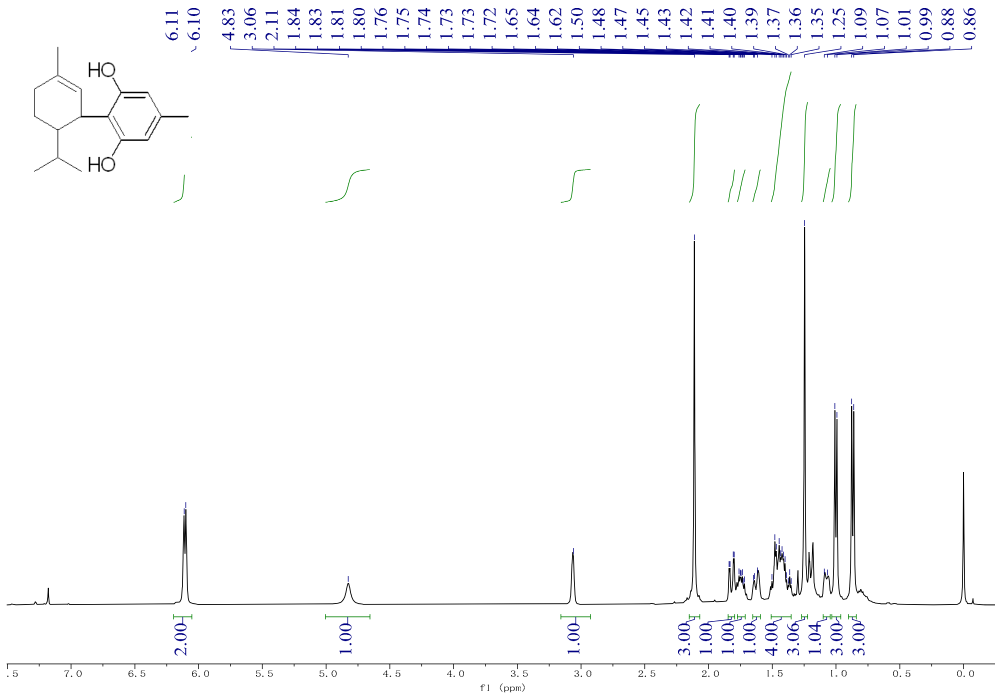
6.11
6.10
4.83
3.06
2.11
1.84
1.83
1.81
1.80
1.76
1.75
1.74
1.73
1.73
1.72
1.65
1.64
1.62
1.50
1.48
1.47
1.45
1.43
1.42
1.41
1.40
1.39
1.37
1.36
1.35
1.25
1.09
1.07
1.01
0.99
0.88
0.86
HO
HO
2.00
1.00
1.00
3.00
1.00
1.00
1.00
4.00
3.06
1.04
3.00
3.00
f1 (ppm)

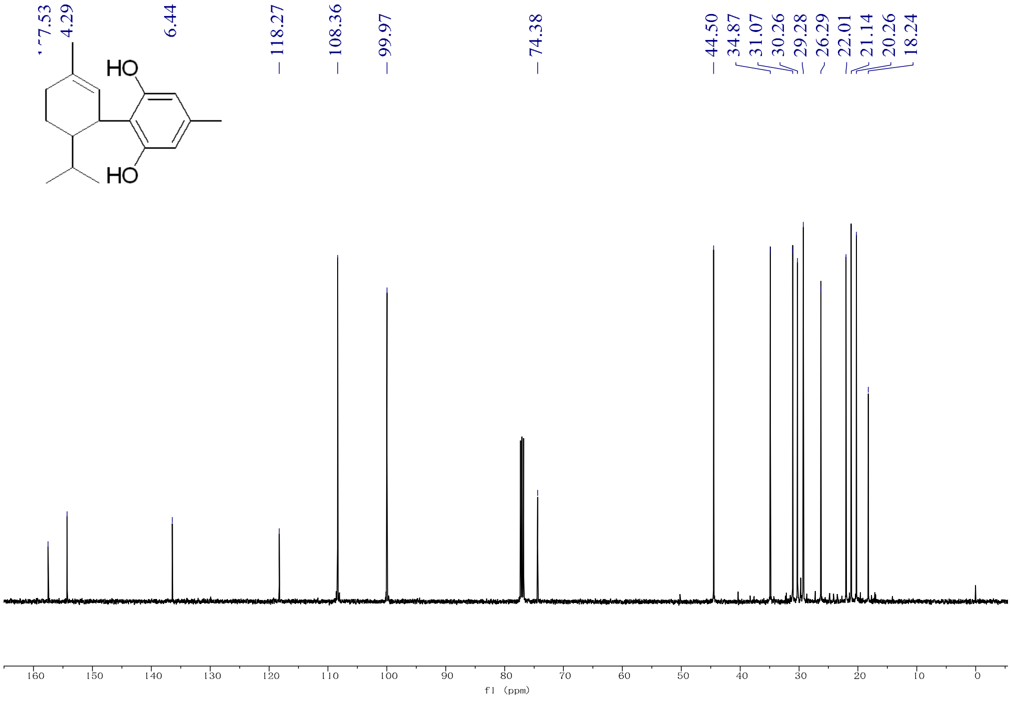
29.28
26.29
22.01
21.14
20.26
18.24
118.27
108.36
99.97
74.38
44.50
34.87
31.07
30.26
HO
HO
f1 (ppm)

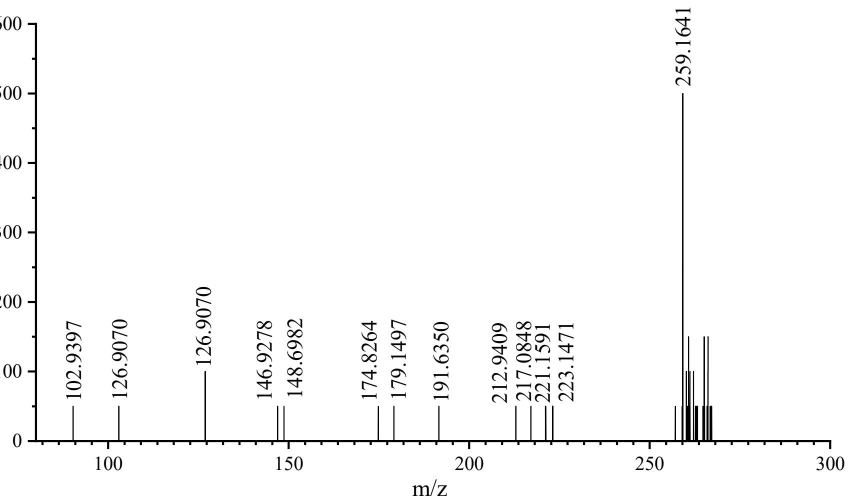

Figure S3. ^{1}H NMR (400 MHz, $CDCl_3$) and ^{13}C NMR(126 MHz, $CDCl_3$) and MS spectra of 2'-Isopropyl-4,5'-dimethyl-1',2'-dihydro-3',4'-tetrahydro-[1,1'-biphenyl]-2,6-diol (**1a**)

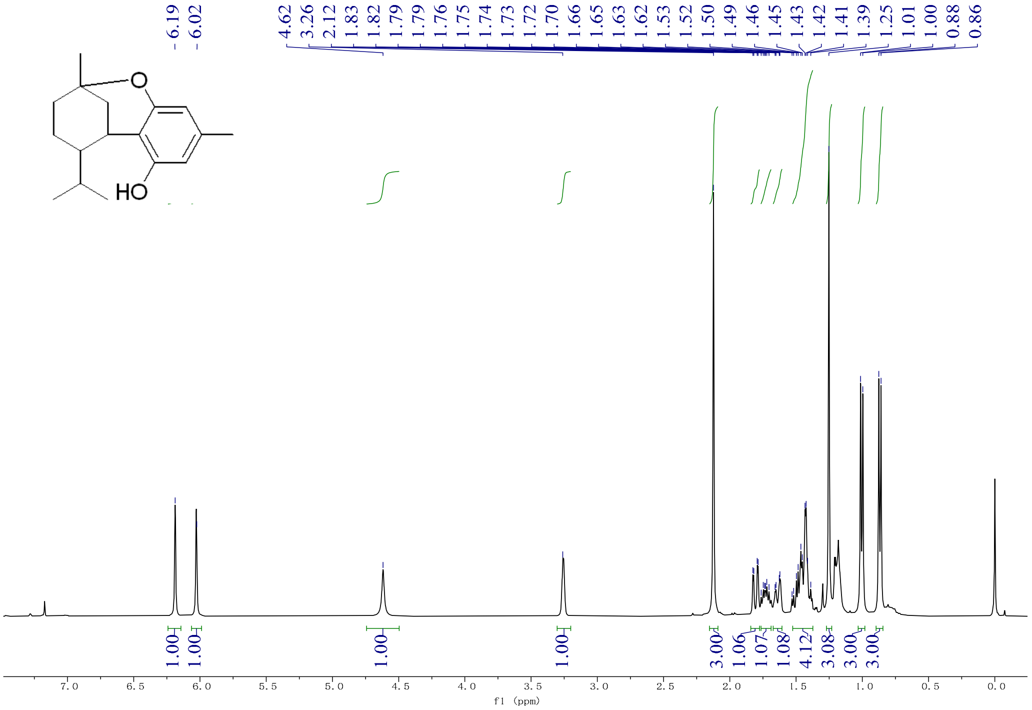
O
HO
6.19
6.02
4.62
3.26
2.12
1.83
1.82
1.79
1.79
1.76
1.75
1.74
1.73
1.72
1.70
1.66
1.65
1.63
1.62
1.53
1.52
1.50
1.49
1.46
1.45
1.43
1.42
1.41
1.39
1.25
1.01
1.00
0.88
0.86
1.00
1.00
1.00
1.00
3.00
1.06
1.07
1.08
4.12
3.08
3.00
3.00
7.0
6.5
6.0
5.5
5.0
4.5
4.0
3.5
3.0
2.5
2.0
1.5
1.0
0.5
0.0
f1 (ppm)

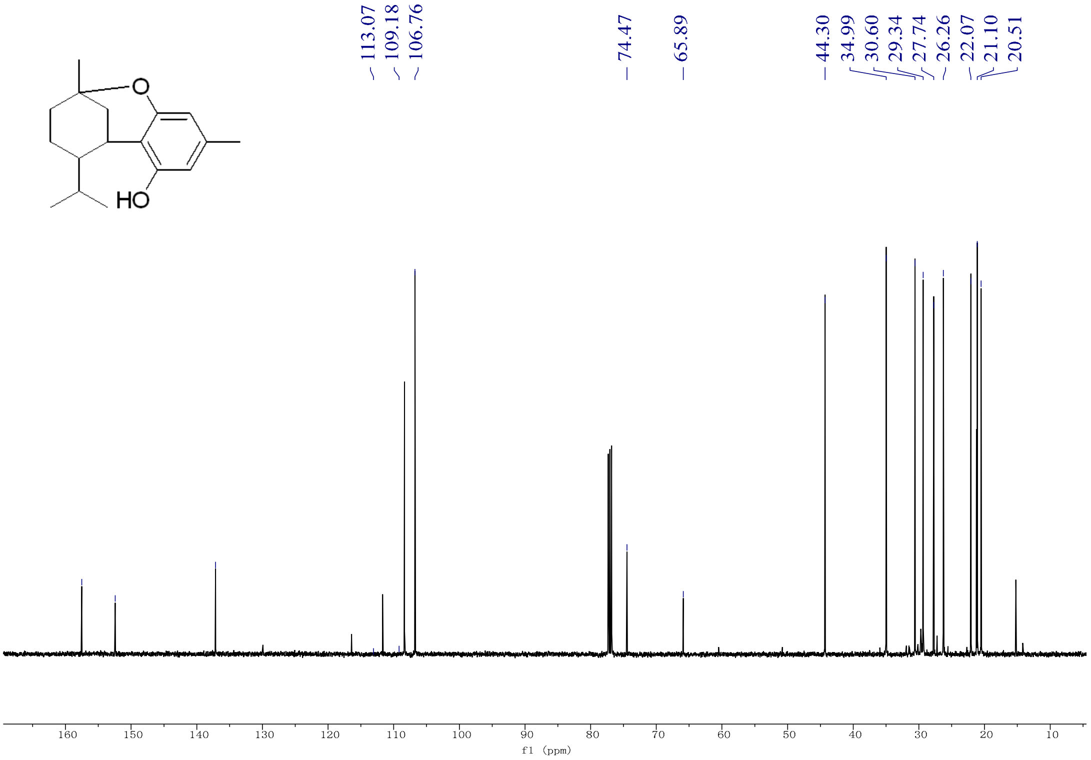
O
HO
113.07
109.18
106.76
74.47
65.89
44.30
34.99
30.60
29.34
27.74
26.26
22.07
21.10
20.51
160
150
140
130
120
110
100
90
80
70
60
50
40
30
20
10
f1 (ppm)

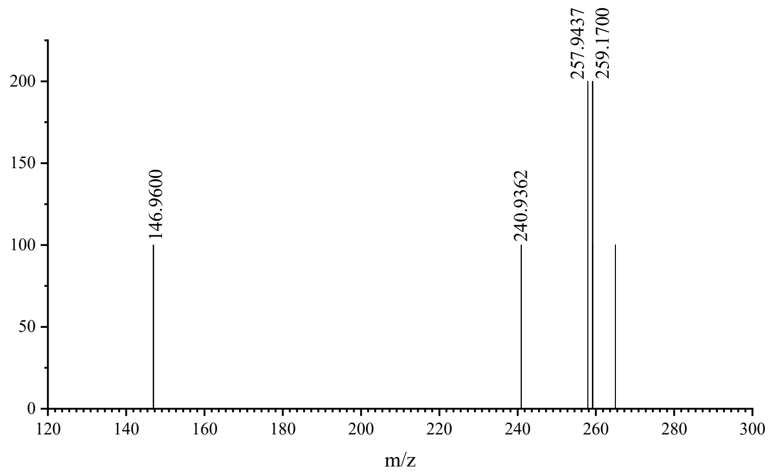

Figure S4. ^{1}H NMR(400 MHz, CDCl3) and ^{13}C NMR(126 MHz, CDCl3) spectra of 2'-Isopropyl-4,5'-dimethyl-1',2'-dihydro-3',4',6'-hexahydro-1',2-methoxybenzo[b]oxacyclooctatrien-6-ol (**1b**)

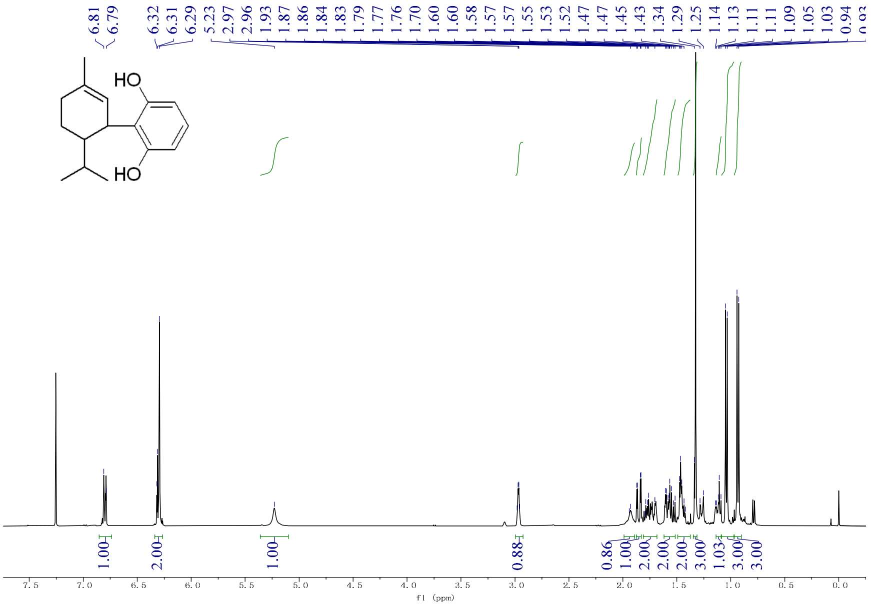

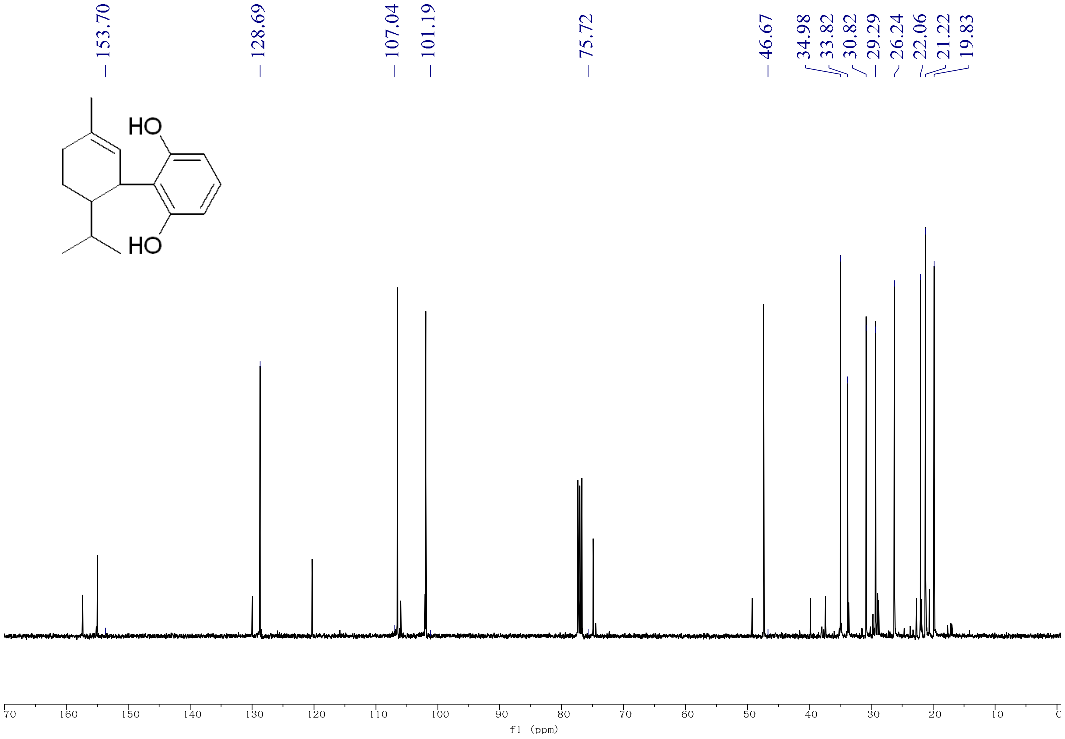

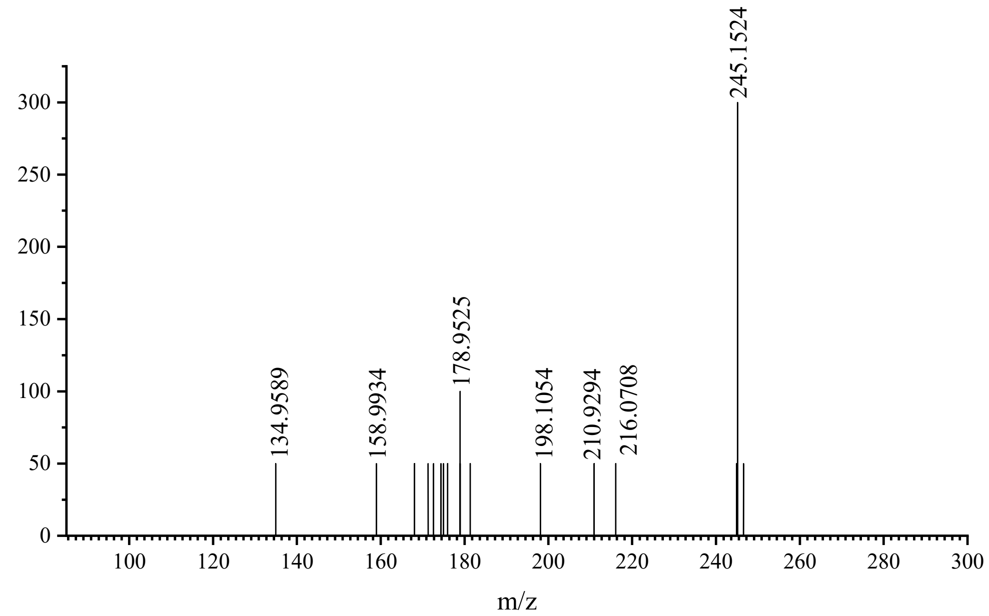

Figure S5. ^{1}H NMR(400 MHz, $CDCl_3$) and ^{13}C NMR(126 MHz, $CDCl_3$) spectra of 2'-Isopropyl-5'-methyl-1',2'-dihydro-3',4'-tetrahydro-[1,1'-biphenyl]-2,6-diol (**2a**)

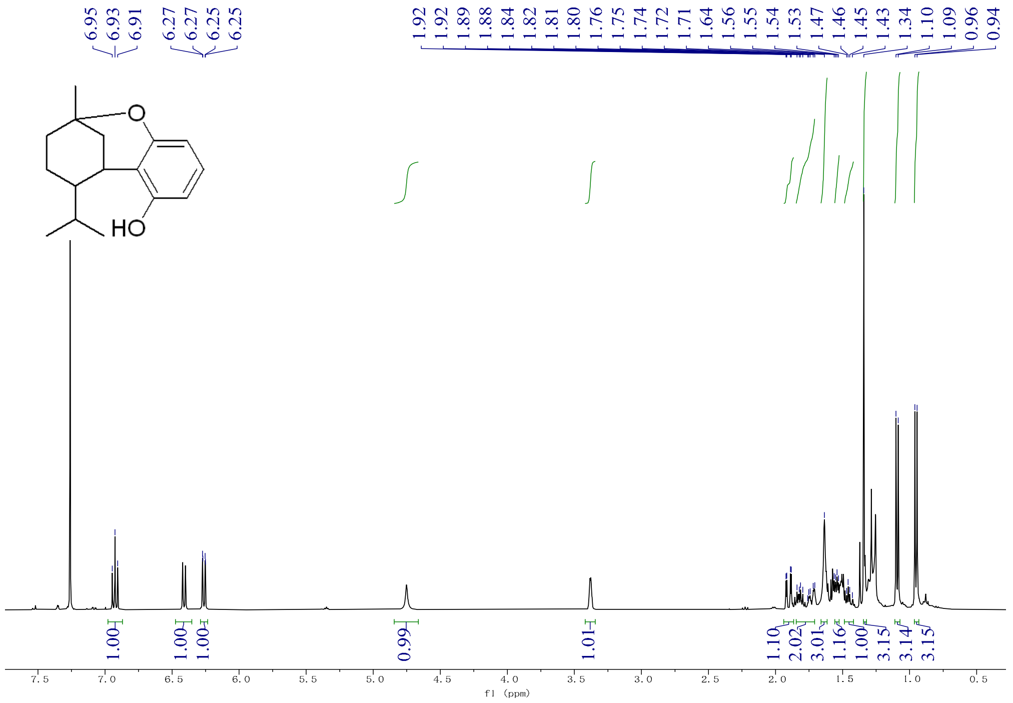
6.95
6.93
6.91
6.27
6.27
6.25
6.25
1.92
1.92
1.89
1.88
1.84
1.82
1.81
1.80
1.76
1.75
1.74
1.72
1.71
1.64
1.56
1.55
1.54
1.53
1.47
1.46
1.45
1.43
1.34
1.10
1.09
0.96
0.94
O
HO
1.00
1.00
1.00
0.99
1.01
1.10
2.02
3.01
1.16
1.00
3.15
3.14
3.15
f1 (ppm)

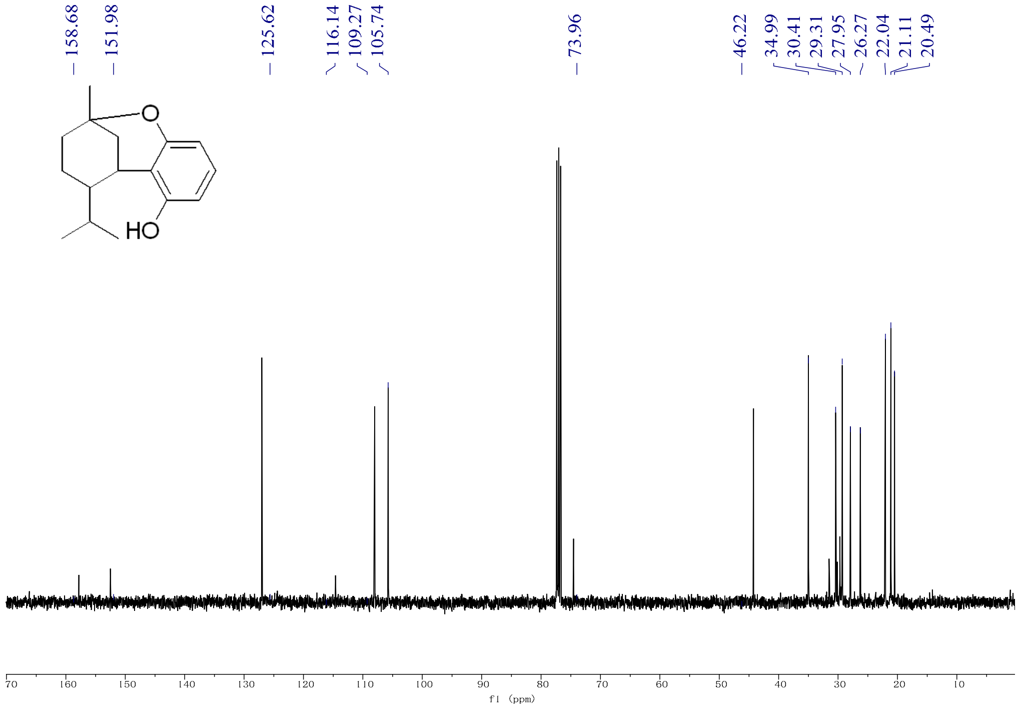
158.68
151.98
125.62
116.14
109.27
105.74
73.96
46.22
34.99
30.41
29.31
27.95
26.27
22.04
21.11
20.49
O
HO
f1 (ppm)

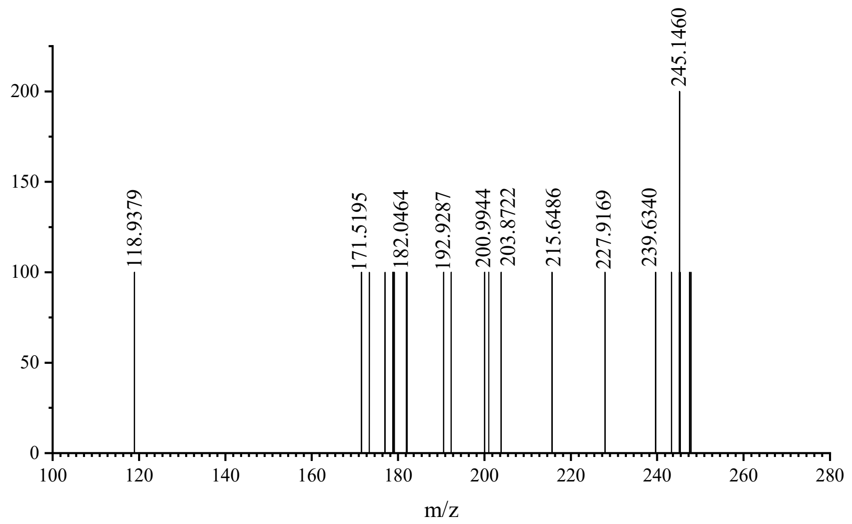

Figure S6. ^{1}H NMR(400 MHz, $CDCl_3$) and ^{13}C NMR(126 MHz, $CDCl_3$) spectra of 2'-Isopropyl-5'-methyl-1',2'-dihydro-3',4',6'-hexahydro-1',2-methoxybenzo[b]oxacyclooctatrien-6-ol (**2b**)

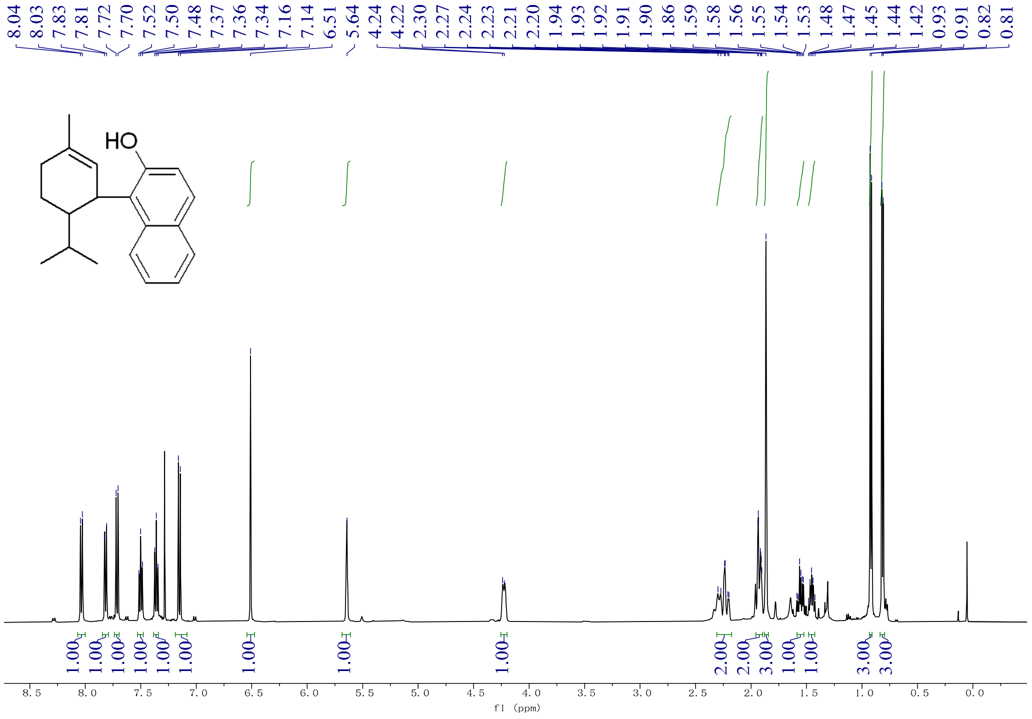
8.04
8.03
7.83
7.81
7.72
7.70
7.52
7.50
7.48
7.37
7.36
7.34
7.16
7.14
6.51
5.64
4.24
4.22
2.30
2.27
2.24
2.23
2.21
2.20
1.94
1.93
1.92
1.91
1.90
1.86
1.59
1.58
1.56
1.55
1.54
1.53
1.48
1.47
1.45
1.44
1.42
0.93
0.91
0.82
0.81
HO
1.00
1.00
1.00
1.00
1.00
1.00
1.00
1.00
1.00
2.00
2.00
3.00
1.00
1.00
3.00
3.00
f1 (ppm)

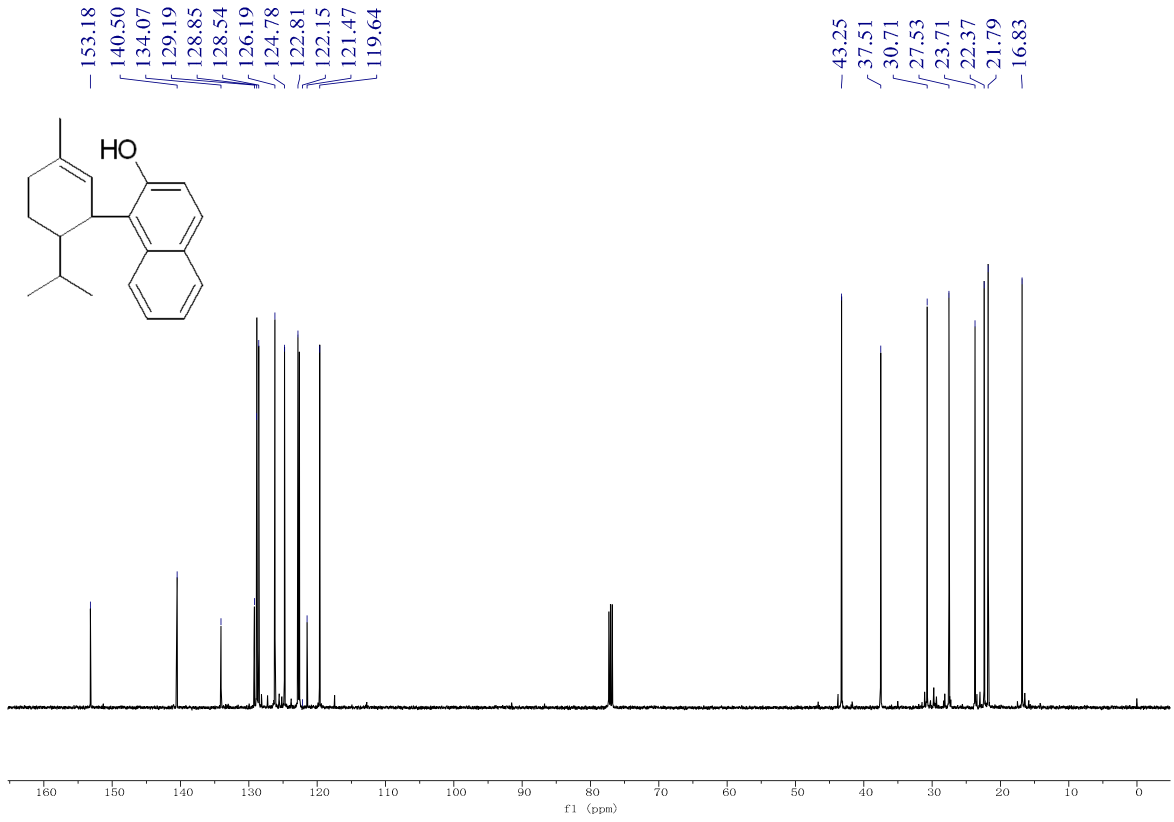
153.18
140.50
134.07
129.19
128.85
128.54
126.19
124.78
122.81
122.15
121.47
119.64
43.25
37.51
30.71
27.53
23.71
22.37
21.79
16.83
HO
f1 (ppm)

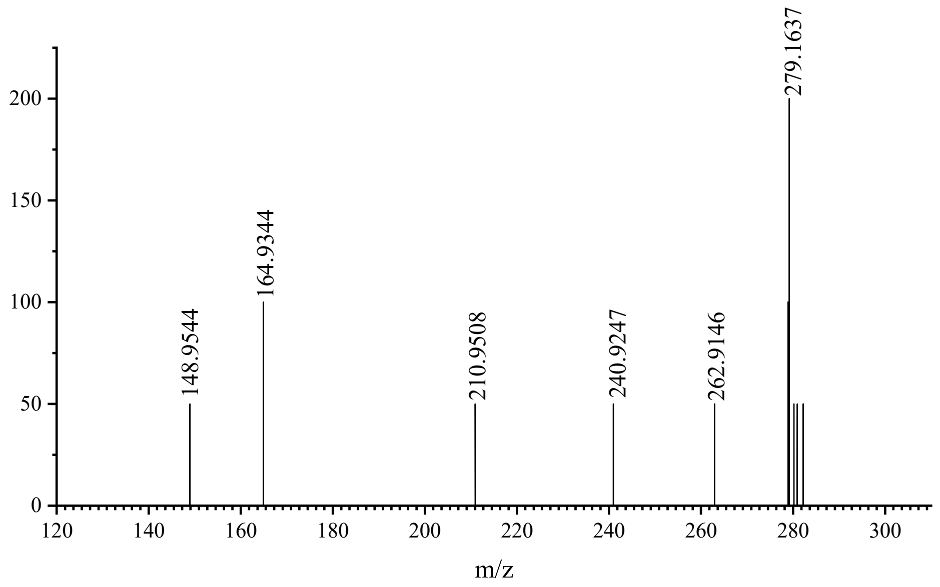

Figure S7. ^{1}H NMR(400 MHz, $CDCl_3$) and ^{13}C NMR(126 MHz, $CDCl_3$) spectra of 2'-Isopropyl-5'-methyl-1',2'-dihydro-3',4'-tetrahydrocyclohexyl-6-en-1-yl)naphthalene-2-ol (**3a**)

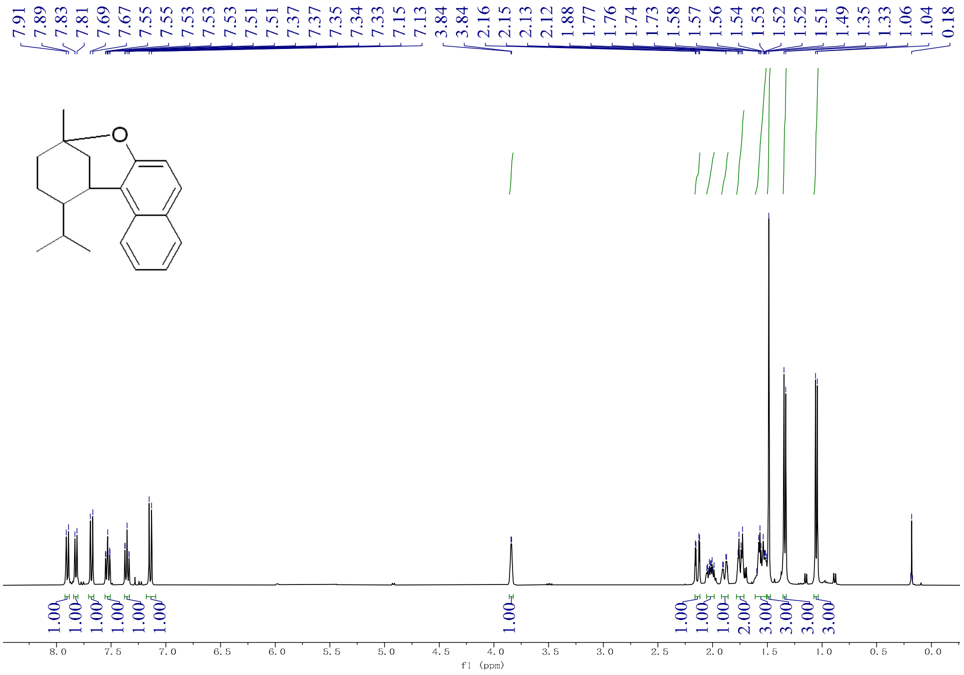
7.91 7.89 7.83 7.81 7.69 7.67 7.55 7.55 7.53 7.53 7.53 7.51 7.51 7.37 7.37 7.35 7.34 7.33 7.15 7.13 3.84 3.84 2.16 2.15 2.13 2.12 1.88 1.77 1.76 1.74 1.73 1.58 1.57 1.56 1.54 1.53 1.52 1.52 1.51 1.49 1.35 1.33 1.06 1.04 0.18
O
1.00 1.00 1.00 1.00 1.00 1.00 1.00 1.00 1.00 1.00 2.00 3.00 3.00 3.00
8.0 7.5 7.0 6.5 6.0 5.5 5.0 4.5 4.0 3.5 3.0 2.5 2.0 1.5 1.0 0.5 0.0
f1 (ppm)

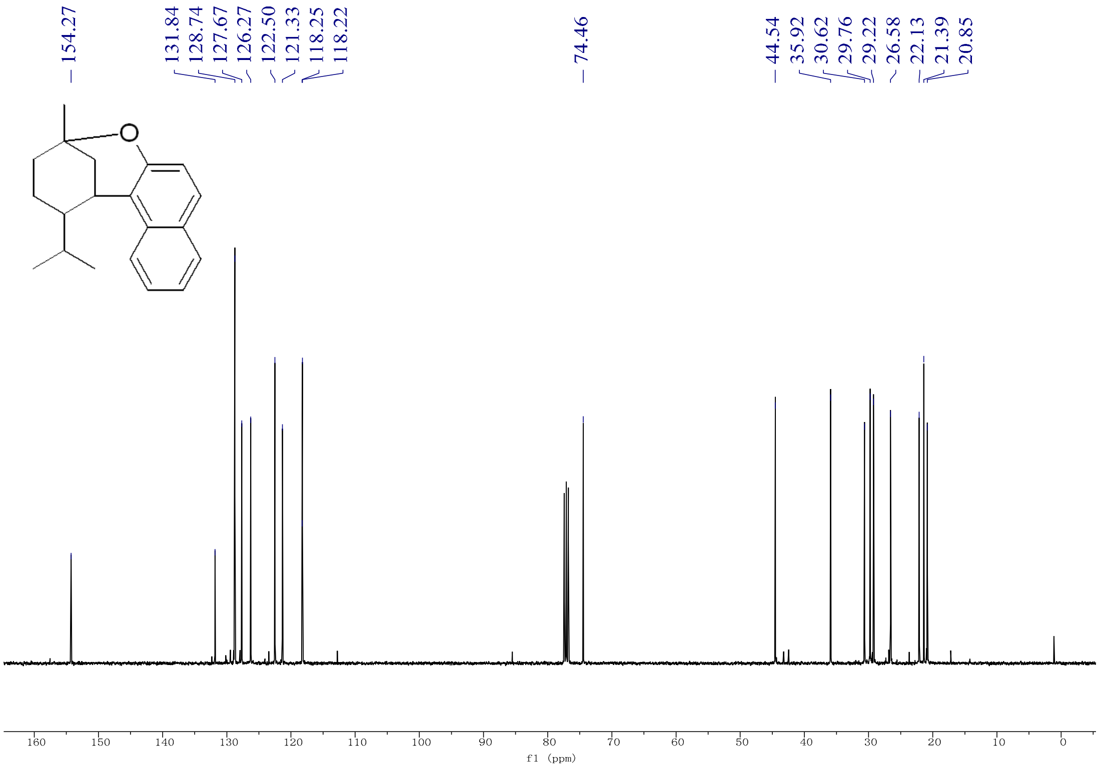
154.27
131.84 128.74 127.67 126.27 122.50 121.33 118.25 118.22
74.46
44.54 35.92 30.62 29.76 29.22 26.58 22.13 21.39 20.85
O
160 150 140 130 120 110 100 90 80 70 60 50 40 30 20 10 0
f1 (ppm)

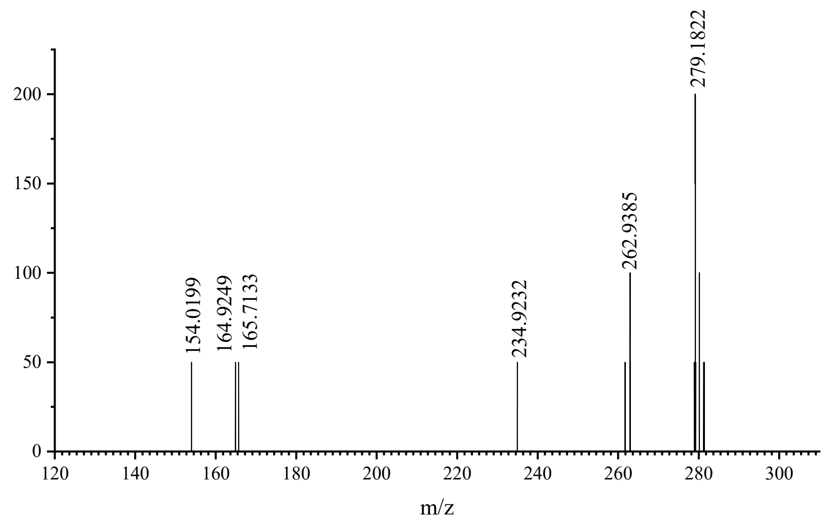

Figure S8. ^{1}H NMR(400 MHz, $CDCl_3$) and ^{13}C NMR(126 MHz, $CDCl_3$) spectra of 2'-Isopropyl-5'-methyl-1',2'-dihydro-3',4',6'-hexahydro-1,7'-methylnonaphthalene[2,1-b]oxoxine (**3b**)

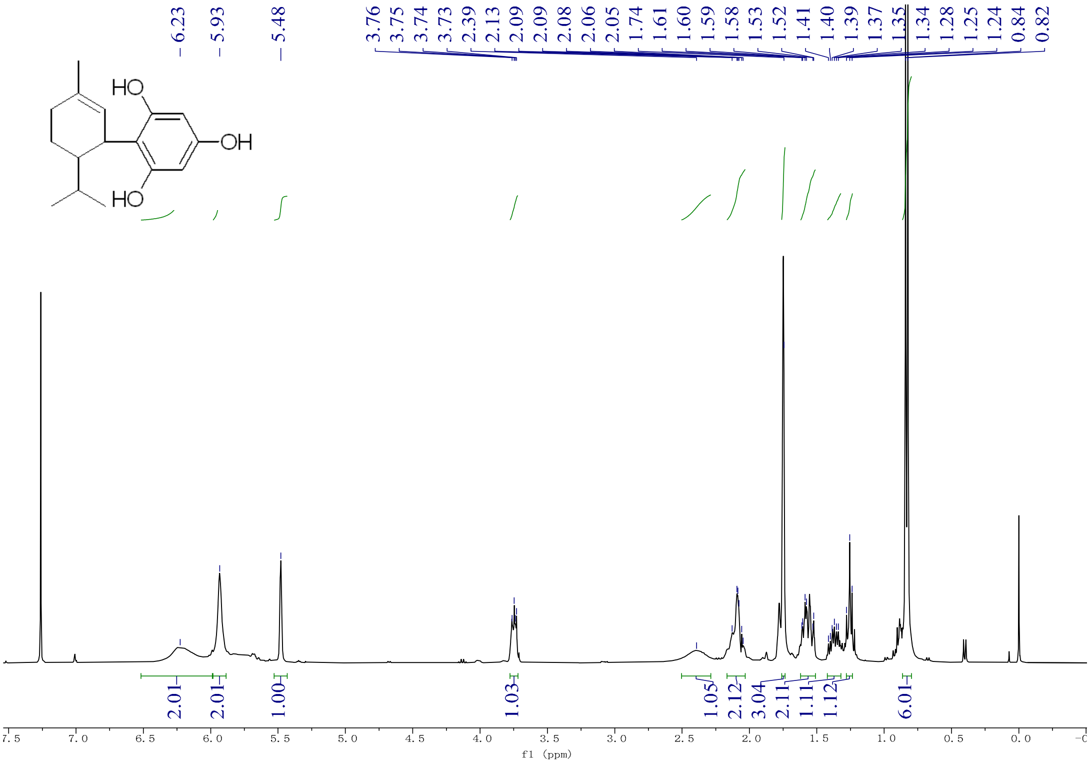
6.23
5.93
5.48
3.76
3.75
3.74
3.73
2.39
2.13
2.09
2.09
2.08
2.06
2.05
1.74
1.61
1.60
1.59
1.58
1.53
1.52
1.41
1.40
1.39
1.37
1.35
1.34
1.28
1.25
1.24
0.84
0.82
HO
OH
HO
2.01
2.01
1.00
1.03
1.05
2.12
3.04
2.11
1.11
1.12
6.01
f1 (ppm)

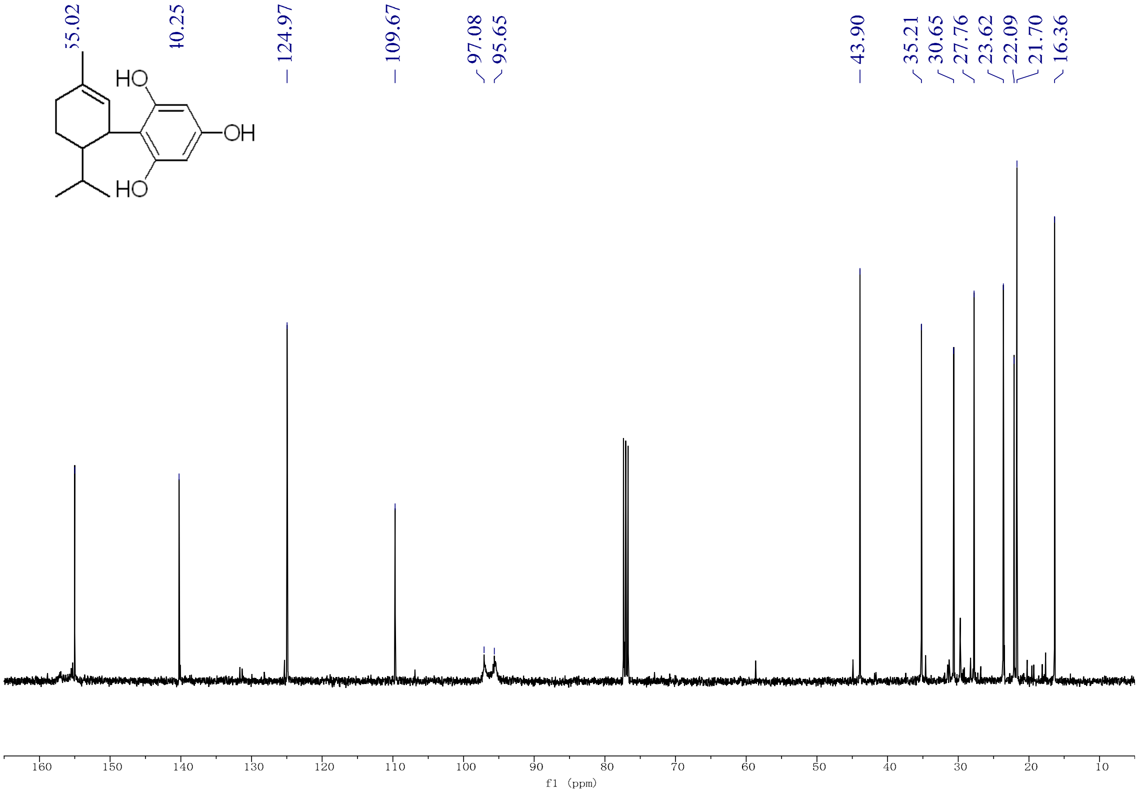
55.02
40.25
124.97
109.67
97.08
95.65
43.90
35.21
30.65
27.76
23.62
22.09
21.70
16.36
HO
OH
HO
f1 (ppm)

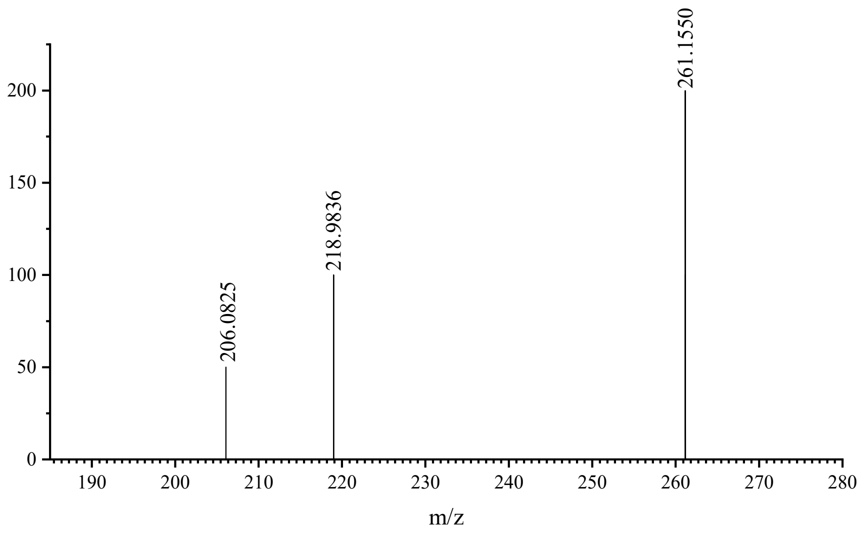

Figure S9. ^{1}H NMR(400 MHz, $CDCl_3$) and ^{13}C NMR(126 MHz, $CDCl_3$) spectra of 2'-Isopropyl-5'-methyl-1',2'-dihydro-3',4'-tetrahydro-[1,1'-biphenyl]-2,4,6-triol (**4**)

H_2CBD

^{1}H NMR (400 MHz, $CDCl_3$) δ 6.23 (s, 3H, 3-H, 5-H, 2-OH), 5.54 (dt, *J* = 2.9, 1.7 Hz, 1H, 6-OH), 4.81 (s, 1H, 6′-H), 3.92~3.77 (m, 1H, 1′-H), 2.52~2.43 (m, 2H, 1″-H), 2.22~2.07 (m, 2H, 4′-H), 1.87~1.76 (m, 4H, 3'-H, 2'-H, 8'-H), 1.72~1.56 (m, 4H, 2''-H, 3''-H), 1.46~1.27 (m, 5H, 4''-H, 5''-H), 0.90 (dt, *J* = 15.3, 6.7 Hz, 9H, 7'-methyl, 9'-methyl, 10'-methyl). **^{13}C NMR** (126 MHz, $CDCl_3$) δ 155.5 (C-2, C-6), 142.9 (C-4), 140.0 (C-5'), 124.8 (C-6'), 114.0 (C-1), 109.7 (C-3), 107.4 (C-5), 43.7 (C-1'), 35.6 (C-2'), 35.5 (C-1''), 31.6 (C-2''), 30.7 (C-3''), 27.8 (C-10'), 24.1 (C-7'), 22.6 (C-4''), 22.1 (C-8'), 21.7 (C-3'), 16.4 (C-9'), 14.1 (C-5''). **MS(EI):** *m/z* 316 $[M]^+$ (15%), 273 $[M\text{-}C_3H_7]^+$ (7%), 260 $[M\text{-}C_4H_8]^+$ (6%), 246$[M\text{-}C_5H_{10}]^+$ (25%),231 $[M\text{-}C_6H_{13}]^+$ (100%), 193 $[M\text{-}C_9H_{15}]^+$ (10%).

6,6,8,9-THC

^{1}H NMR (400 MHz, $CDCl_3$) δ 6.31 (s, 1H, 3-H), 6.14 (s, 1H, 5-H), 4.80 (s, 1H, 6-OH), 3.37 (s, 1H, 1′-H), 2.50~2.45 (m, 2H, 1″-H), 1.94~1.82 (m, 2H, 4′-H), 1.76 (dq, *J* = 13.3, 3.2 Hz, 1H, 2′-H), 1.62~1.53 (m, 5H, 3'-H, 6'-H, 8'-H), 1.38~1.29 (m, 9H, 2''-H, 3''-H, 4''-H, 7'-methyl), 1.12 (d, *J* = 6.6 Hz, 3H, 9'-methyl), 0.98 (d, *J* = 6.5 Hz, 3H, 10'-methyl), 0.92 (t, *J* = 6.8 Hz, 3H, 5''-H). **^{13}C NMR** (126 MHz, $CDCl_3$) δ 157.5 (C-2), 152.1 (C-6), 142.4 (C-4), 111.8 (C-1), 107.8 (C-5), 106.2 (C-3), 74.5 (C-5'), 44.3 (C-1'), 35.7 (C-4'), 35.0 (C-3'), 31.6 (C-6'), 30.8 (C-3''), 30.6 (C-4''), 29.4 (C-7'), 27.8 (C-8'), 26.3 (C-2'), 22.6 (C-2''), 22.1 (C-10'), 21.1 (C-9'), 20.6 (C-1''), 14.1 (C-5''). **MS(EI):** *m/z* 316 $[M]^+$ (15%), 246$[M\text{-}C_5H_{10}]^+$ (25%),231 $[M\text{-}C_6H_{13}]^+$ (100%), 203 $[M\text{-}C_8H_{17}]^+$ (10%), 189$[M\text{-}C_9H_{19}]^+$ (10%).

1a: 2'-Isopropyl-4,5'-dimethyl-1',2'-dihydro-3',4'-tetrahydro-[1,1'-biphenyl]-2,6-diol

^{1}H NMR (400 MHz, $CDCl_3$) δ 6.11 (d, *J* = 5.1 Hz, 2H, 3-H, 5-H), 4.83 (s, 1H, 6′-H), 3.06 (s, 1H, 1′-H), 2.11 (s, 3H, 1″-H), 1.82 (dd, *J* = 13.2, 2.8 Hz, 1H, 2′-H), 1.77~1.71 (m, 1H), 1.65~1.59 (m, 1H), 1.51~1.35 (m, 4H, 4′-H, 3′-H), 1.25 (s, 3H, 7′-methyl), 1.08 (d, *J* = 10.2 Hz, 1H, 8′-H), 1.00 (d, *J* = 6.6 Hz, 3H, 9′-methyl), 0.87 (d, *J* = 6.5 Hz, 3H, 10′-methyl). **^{13}C NMR** (126 MHz, $CDCl_3$) δ 157.53 (C-4), 154.29 (C-2, C-6), 136.44 (C-5′), 118.27 (C-6′), 108.36 (C-1), 99.97 (C-3, C-5), 74.38(C-1″), 44.50 (C-1′), 34.87 (C-2′), 30.26 (C-4′), 29.28, 26.29 (C-8′), 22.01 (C-3′), 21.14 (C-7′), 18.24 (C-9′, C-10′). **HRMS (ESI-TOF)** *m/z* 260.1641 $[M-H]^-$ (calcd for $C_{17}H_{24}O_2$, 260.1776).

1b: 2'-Isopropyl-4,5'-dimethyl-1',2'-dihydro-3',4',6'-hexahydro-1',2-methoxybenzo[b]oxacyclooctatrien-6-ol

^{1}H NMR (400 MHz, $CDCl_3$) δ 6.19 (s, 1H, 3-H), 6.02 (s, 1H, 5-H), 4.62 (s, 1H, 6-OH), 3.26 (s, 1H, 1′-H), 2.12 (s, 3H, 1″-H), 1.81 (dd, *J* = 13.2, 2.7 Hz, 1H, 2′-H), 1.73 (dt, *J* = 10.6, 6.5 Hz, 1H), 1.64 (dd, *J* = 12.1, 2.6 Hz, 1H, 8′-H), 1.53~1.37 (m, 4H, 4′-H, 3′-H), 1.25 (s, 3H, 7′-methyl), 1.21~1.16 (m, 2H, 6′-H), 1.00 (d, *J* = 6.6 Hz, 3H, 9′-methyl), 0.87 (d, *J* = 6.5 Hz, 3H, 10′-methyl). **^{13}C NMR** (126 MHz, $CDCl_3$) δ 157.51 (C-1), 152.43 (C-2), 137.13 (C-4), 113.07 (C-6), 109.18 (C-3), 106.76 (C-5), 74.47 (C-1″), 65.89 (C-1′), 44.30 (C-4′), 34.99 (C-6′), 30.60 (C-8′), 29.34

(C-2′), 27.74 (C-7′), 26.26 (C-5′), 22.07 (C-3′), 20.51 (C-9′, C-10′). **HRMS** (ESI-TOF) *m/z* 260.1700 $[M - H]^-$ (calcd for $C_{17}H_{24}O_2$, 260.1810).

2a: 2'-Isopropyl-5'-methyl-1',2'-dihydro-3',4'-tetrahydro-[1,1'-biphenyl]-2,6-diol

^{1}**H NMR** (400 MHz, $CDCl_3$) δ 6.80 (d, *J* = 9.6 Hz, 1H, 4-H), 6.34~6.26 (m, 2H, 3-H, 5-H), 5.23 (s, 1H, 6′-H), 2.97 (q, *J* = 3.1 Hz, 1H, 1′-H), 1.93 (br, *J* = 4.4 Hz, 1H, 2-OH), 1.85 (dd, *J* = 13.2, 2.6 Hz, 1H, 2′-H), 1.77 (d, *J* = 3.7 Hz, 1H, 8′-H), 1.62~1.51 (m, 2H, 4′-H), 1.49~1.38 (m, 2H, 3′-H), 1.34 (s, 3H, 7′-methyl), 1.14~1.09 (m, 1H, 6-OH), 1.04 (d, *J* = 6.6 Hz, 3H, 9′-methyl), 0.93 (d, *J* = 6.5 Hz, 3H, 10′-methyl). ^{13}**C NMR** (126 MHz, $CDCl_3$) δ 153.70 (C-2, C-6), 128.69 (C-4), 120.31 (C-1), 107.04 (C-3, C-5), 101.19(C-6′), 75.72 (C-5′), 46.67 (C-1′), 34.98 (C-2′), 30.82 (C-4′), 26.24 (C-8′), 22.06 (C-3′), 21.22 (C-7′), 19.83 (C-9′, C-10′). **HRMS** (ESI-TOF) *m/z* 246.1524 $[M - H]^-$ (calcd for $C_{17}H_{24}O_2$, 246.1620).

2b: 2'-Isopropyl-5'-methyl-1',2'-dihydro-3',4',6'-hexahydro-1',2-methoxybenzo[b]oxacyclooctatrien-6-ol

^{1}**H NMR** (400 MHz, $CDCl_3$) δ 6.93 (t, *J* = 8.1 Hz, 1H, 4-H), 6.41 (dd, *J* = 8.3, 1.0 Hz, 1H, 5-H), 6.26 (dd, *J* = 7.9, 1.0 Hz, 1H, 3-H), 4.75 (s, 1H, 6-OH), 3.38 (q, *J* = 3.1 Hz, 1H, 1′-H), 1.90 (dd, *J* = 13.3, 2.7 Hz, 1H, 2′-H), 1.85~1.71 (m, 2H, 4′-H), 1.64 (s, 3H, 8′-H, 3′-H), 1.54 (d, *J* = 4.3 Hz, 2H, 6′-H), 1.34 (s, 3H, 7′-methyl), 1.09 (d, *J* = 6.6 Hz, 3H, 9′-methyl), 0.95 (d, *J* = 6.5 Hz, 3H, 10′-methyl). ^{13}**C NMR** (126 MHz, $CDCl_3$) δ 158.68 (C-1), 151.98 (C-2), 125.62 (C-4), 116.14 (C-6), 109.27 (C-3), 105.74 (C-5), 73.96 (C-5′), 46.22 (C-1′), 34.99 (C-2′), 30.41 (C-4′), 29.31 (C-6′), 26.27 (C-8′), 22.04 (C-3′), 21.11 (C-7′), 20.49 (C-9′, C-10′). **HRMS** (ESI-TOF) *m/z* 246.1460 $[M - H]^-$ (calcd for $C_{17}H_{24}O_2$, 246.1653).

3a: 2'-Isopropyl-5'-methyl-1',2'-dihydro-3',4'-tetrahydrocyclohexyl-6-en-1-yl)naphthalene-2-ol

^{1}**H NMR** (400 MHz, $CDCl_3$) δ 8.04 (d, *J* = 8.7 Hz, 1H, 9-H), 7.82 (d, *J* = 9.6 Hz, 1H, 6-H), 7.71(d, *J* = 8.8 Hz, 1H, 4-H), 7.50 (t, *J* = 8.5 Hz, 1H, 7-H), 7.36 (t, *J* = 7.5 Hz, 1H, 8-H), 7.15 (d, *J* = 8.8 Hz, 1H, 3-H), 6.51 (s, 1H, 2-OH), 5.64 (s, 1H, 6′-H), 4.23 (d, *J* = 7.5 Hz, 1H, 1′-H), 2.31~2.17 (m, 2H, 4′-H), 1.92 (dd, *J* = 16.1, 2.5 Hz, 2H, 3′-H), 1.86 (s, 3H, 7′-methyl), 1.55 (dt, *J* = 12.0, 6.3 Hz, 1H, 2′-H), 1.48~1.42 (m, 1H, 8′-H), 0.92 (d, *J* = 6.9 Hz, 3H, 9′-methyl), 0.81 (d, *J* = 7.0 Hz, 3H, 10′-methyl). ^{13}**C NMR** (126 MHz, $CDCl_3$) δ 238.47 (C-2), 222.35 (C-10), 153.18 (C-5), 140.50 (C-4), 134.07 (C-8), 129.19 (C-1), 128.85 (C-9), 126.19 (C-7), 124.78 (C-3), 122.81 (C-6), 122.15 (C-6′), 121.47 (C-5′), 119.64 (C-1′), 43.25 (C-2′), 37.51 (C-4′), 30.71 (C-8′), 27.53 (C-3′), 23.71 (C-7′), 22.37 (C-9′), 21.79 (C-10′). **HRMS** (ESI-TOF) *m/z* 280.1637 $[M - H]^-$ (calcd for $C_{17}H_{24}O_2$, 280.1827).

3b: 2'-Isopropyl-5'-methyl-1',2'-dihydro-3',4',6'-hexahydro-1,7'-methylnonaphthalene[2,1-b]oxoxine

^{1}**H NMR** (400 MHz, $CDCl_3$) δ 7.90 (d, *J* = 8.5 Hz, 1H, 9-H), 7.82 (d, *J* = 8.0 Hz, 1H, 6-H), 7.68 (d, *J* = 8.9 Hz, 1H, 4-H), 7.53 (ddd, *J* = 8.4, 6.9, 1.3 Hz, 1H, 7-H), 7.38~7.33 (m, 1H, 8-H), 7.14 (d, *J* = 8.9 Hz, 1H, 3-H), 3.86~3.82 (m, 1H, 1′-H), 2.14~2.02 (ddt, *J* = 13.1, 6.6, 3.4 Hz, 1H, 4′-H), 1.92~1.86 (m, 1H, 2′-H), 1.78~1.71 (m, 2H, 3′-H), 1.61~1.51 (m, 3H, 6′-H, 8′-H), 1.49 (s, 3H, 7′-methyl), 1.34 (d, *J* = 6.6 Hz, 3H, 9′-methyl), 1.05 (d, *J* = 6.6 Hz, 3H, 10′-methyl). ^{13}**C NMR** (126 MHz, $CDCl_3$) δ 154.27(C-2), 131.84(C-10), 128.74(C-5), 127.67(C-4), 126.27(C-8), 122.50(C-1), 121.33(C-9), 118.25(C-7), 118.22(C-3), 112.78(C-6), 74.46 (C-1′), 44.54 (C-2′), 35.92 (C-4′), 30.62 (C-8′), 29.76 (C-3′), 29.22 (C-5′), 26.58 (C-6′), 22.13 (C-7′), 21.39 (C-9′), 20.85 (C-10′). **HRMS** (ESI-TOF) *m/z* 280.1822 $[M - H]^-$ (calcd for $C_{17}H_{24}O_2$, 280.1861).

4: 2'-Isopropyl-5'-methyl-1',2'-dihydro-3',4'-tetrahydro-[1,1'-biphenyl]-2,4,6-triol

^{1}**H NMR** (400 MHz, $CDCl_3$), δ: 6.23 (s, 2H, 3-H, 5-H), 5.93 (s, 2H, 2-OH, 6-OH), 5.48 (s, 1H, 6′-H), 3.74 (dd, *J* = 8.1、5.2 Hz, 1H, 1′-H), 2.39 (br s, 1H, 4-OH), 2.17~2.03 (m, 2H, 4′-H), 1.74 (s, 3H, 7′-methyl), 1.62~1.51 (m, 2H, 3′-H), 1.42~1.32 (m, 1H, 2′-H), 1.28~1.24 (m, 1H, 8′-H), 0.83 (d, *J* = 6.8 Hz, 6H, 9′-methyl, 10′-methyl).^{13}**C NMR** (126 MHz, $CDCl_3$), δ: 155.02 (C-4), 140.25 (C-2, C-6), 124.97 (C-5′), 109.67 (C-6′), 97.08 (C-1), 95.65 (C-3, C-5), 43.90 (C-2′), 35.21 (C-1′), 30.65 (C-4′), 27.76 (C-8′), 23.62 (C-3′), 22.09 (C-7′), 21.70 (C-9′, C-10′). **HRMS** (ESI-TOF) *m/z* 262.1550 $[M - H]^-$ (calcd for $C_{17}H_{24}O_2$, 262.1569).
